# Concentrations of persistent organic pollutants in maternal and cord blood from the maternal-infant research on environmental chemicals (MIREC) cohort study

**DOI:** 10.1186/s12940-016-0143-y

**Published:** 2016-05-04

**Authors:** Mandy Fisher, Tye E. Arbuckle, Chun Lei Liang, Alain LeBlanc, Eric Gaudreau, Warren G. Foster, Douglas Haines, Karelyn Davis, William D. Fraser

**Affiliations:** Healthy Environments and Consumer Safety Branch, Health Canada, Ottawa, ON Canada; Le Centre de toxicologie du Québec, Institut nationale de santé publique du Québec, Québec, QC Canada; Department of Obstetrics and Gynecology, Division of Reproductive Biology, McMaster University, Hamilton, ON Canada; Centre hospitalier universitaire de Sherbrook (CHUS), Sherbrooke, QC Canada; Population Studies Division, Environmental Health Science and Research Bureau, Environmental and Radiation Health Sciences Directorate, Health Canada, 50 Columbine Driveway, Tunney’s Pasture, Ottawa, AL 0801A K1A 0K9 Canada

**Keywords:** POPs, DDT, PCBs, PBDEs, PFASs, Cord plasma, Maternal plasma, Pregnant

## Abstract

**Background:**

Pregnant women are an especially important population to monitor for environmental exposures given the vulnerability of the developing fetus. During pregnancy and lactation chemical body burdens may change due to the significant physiological changes that occur. Developmental exposures to some persistent organic pollutants (POPs) have been linked with adverse health outcomes.

**Methods:**

First trimester maternal and cord blood plasma concentrations of several POPs including polychlorinated biphenyls (PCBs), organochlorine pesticides (OCs), polybrominated diphenyl ethers (PBDE)s and perfluoroalkyl substances (PFASs) were measured in samples from 1983 pregnant women enrolled in the Maternal-Infant Research on Environmental Chemicals (MIREC) cohort. Predictors of exposure were also identified.

**Results:**

In maternal plasma, there was >90 % detection for the perfluoroalkyl substances (PFASs) perfluorooctanoic acid (PFOA), perfluoroctane sulfonate (PFOS), perfluorohexane sulfonate (PFHxS), and dichlorodiphenyldichloroethylene (DDE), oxychlordane and PCB 138 and 153. Cord blood plasma had much lower detection rates with low or very limited detection for most PCBs and PBDEs. The PFASs were the most frequently detected (23–64 %) chemical class in cord plasma. In a subset of 1st and 3rd trimester paired samples, PFAS concentrations were found to be strongly correlated and had ICCs ranging from 0.64 (PFOA) to 0.83 (PFHxS). The cord:maternal plasma concentration ratios ranged from 0.14 (PFOS) to 0.87 (oxychlordane, lipid adjusted). Similar to other studies, we found parity, maternal age, income, education, smoking status, pre-pregnancy BMI and fish consumption to be significant predictors for most chemicals. Those participants who were foreign-born had significantly higher concentrations of organochlorinated pesticides and PCBs.

**Conclusions:**

In the MIREC study, multiple chemical contaminants were quantified in the plasma of pregnant women. In cord plasma PFOA had the highest detection rate. However, compared to other Canadian and international population studies, the MIREC participants had lower contaminant concentrations of these substances.

**Electronic supplementary material:**

The online version of this article (doi:10.1186/s12940-016-0143-y) contains supplementary material, which is available to authorized users.

## Background

Persistent organic pollutants (POPs), including organochlorine pesticides (OCs), industrial chemicals and their by-products are chemicals that persist in the environment and tend to bioaccumulate, possess toxic properties, and resist degradation [1]. Several biomonitoring studies have measured POPs in human plasma [2–4], milk [5, 6], and cord blood [7–9].

Perfluoroalkyl substances (PFASs) are heat stable, non-flammable and able to repel water and oils. They are used in a wide variety of products including non-stick cookware, breathable all weather clothing, wiper blades, wire and cable insulation, fire retardant foams, lubricants, paper coatings, pharmaceuticals, and nail polish [2]. They are very stable compounds and therefore highly persistent in the environment [10]. In 2002, 3M, the main manufacturer of PFOS ceased production in the U.S. and the EPA finalized new rules to help limit the future manufacturing and importation of these substances [11]. Efforts were implemented in 2006 to drastically reduce emissions of PFOA and its precursors by 95 % by 2010 and to work towards eliminating emissions and product content by 2015 [12]. Exposures to humans mainly occur through diet [13, 14].

PBDEs are a class of brominated flame retardants that are environmentally persistent, bioaccumulate and biomagnify in terrestrial food chains [15]. Studies have found PBDEs in fish, dairy products and eggs [16, 17]. House dust [18, 19] has also been suggested as a significant source of exposure, especially for children [20]. The Government of Canada has placed restrictions on some PBDEs under the Canadian Environmental Protection Act, 1999 in order to protect the environment. As of 2008 regulation prohibit the manufacturing of all PBDEs in Canada, and restrict the import and sale of PBDEs in mixtures [21]. Furthermore, as of 2009 there has been a phasing out of penta- and octa-BDEs had occurred in North America, Europe, Australia and Japan [22].

Pregnant women are an especially important population to include in biomonitoring and epidemiological studies given the susceptibility of the developing fetus [10, 23, 24] to the potential adverse health effects arising from exposure to environmental chemicals. During pregnancy and lactation, body burdens of chemicals may change due to the significant physiological changes that occur. The pregnant woman experiences increased blood volume, enhanced metabolism, increased renal perfusion as well as substantial changes to circulating hormones, essential elements and serum lipids [25, 26]. These changes may have an impact on absorption, distribution, metabolism and excretion of environmental contaminants, which has potentially serious implications for developmental exposures of the fetus. Some POP exposures during fetal developmental have been linked with thyroid hormone disruption [23, 27–29], adverse birth outcomes [30–32], poorer respiratory health [33], obesity [34], and neurotoxicity [35, 36].

This article describes both maternal and cord plasma concentrations of several POPs including polychlorinated biphenyls (PCBs), OC pesticides, polybrominated diphenyl ethers (PBDE)s and perfluoroalkyl substances (PFASs) measured in the Canadian prospective cohort, Maternal-Infant Research on Environmental Chemicals (MIREC). We also explored maternal and infant characteristics to identify elevated exposure groups.

## Methods

The MIREC Study is a national-level pregnancy cohort of approximately 2000 women recruited from 10 cities across Canada and has been previously described [37]. The MIREC study is based on a convenience sample and enrolled pregnant women from the general population who were attending prenatal clinics (ultrasound, midwife and/or doctor’s clinics) during the first trimester of pregnancy (6 to <14 weeks) between 2008 and 2011. Research Ethics Board approval was obtained from all participating sites and Health Canada. Women who gave informed consented scheduled visits in each trimester, at delivery and in the postpartum period (up to 8-10 weeks). At each pregnancy visit women completed questionnaires and provided both blood and urine samples. The questionnaires collected information such as sociodemographics, current and previous pregnancies, diet, smoking and lifestyle. Maternal blood was collected in 10 mL K2 EDTA tubes; plasma was transferred into 2.5 mL pre-cleaned glass vials (Supelco®) for the POPs analysis and a 5 mL Sarstedt® tube for the PFASs analysis. Cord blood was collected at delivery using a Baxter bag® and then transferred into tubes as described above for maternal blood. Samples were stored at -20 °C.

In the MIREC study there were close to 300 chemical metabolites measured in maternal urine, blood, cord blood, meconium and breast milk which have been described elsewhere [37]. These chemicals were chosen based on evidence of potential reproductive toxicity, and valid laboratory methods available for biomarkers. A subset of chemicals was also chosen based on that measured in Cycle 1 (2007–2009) of the Canadian Health Measures Survey [2] so that comparisons could be made to the general population biomonitoring data, given that measurements were made by the same laboratory. Only the persistent organic pollutants and PFASs measured in maternal and cord plasma are reported in this article. A total of 24 polychlorinated biphenyls (PCB 28, 52, 66, 74, 99, 101, 105, 118, 128, 138, 146, 153, 156, 163, 167, 170, 178, 180, 183, 187, 194, 201, 203 and 206), 14 organochlorinated pesticides (aldrin, beta-hexachlorocyclohexane (β-HCH), gamma-hexachlorocyclohexane (γ-HCH), hexachlorobenzene, cis-nonachlor, trans-nonachlor, α-chlordane, γ-chlordane, oxychlordane, dichlorodiphenyldichloroethylene (DDE), p,p’-dichlorodiphenyltrichloroethane (DDT), mirex, toxaphenes Parlar 26 and 50) and 9 polybrominated diphenyl ethers (PBDE 15, 17, 25, 28, 33, 47, 99, 100, 153) and hexabromobiphenyl (PBB 153) were measured by the Centre de Toxicologie du Québec (CTQ) of the Institut National de Santé Publique du Québec (INSPQ) in 1st trimester maternal plasma samples as well as cord plasma samples. The PFASs perfluorooctane sulfonate (PFOS), perfluorooctanoic acid (PFOA) and perfluorohexane sulfonate (PFHxS) were also measured in both 1st trimester maternal and cord plasma. For a subset of participants (*n* = 48) PFASs were also measured in the 3rd trimester.

### Analytical methods

Two mL of plasma was enriched with labeled internal standards (PCB 141-^13^C_12,_ PCB 153, -^13^C_12,_ PCB 180-^13^C_12_, PCB 194-^13^C_12_, hexachlorobenzene-^13^C_6_, α-HCH-^13^C_6_, trans-nonachlor-^13^C_10_, oxychlordane-^13^C_10,_ p,p’-DDE-^13^C_12_, Parlar 26-^13^C_10_, Parlar 50-^13^C_10_, PBDE-77-^13^C_12_) plus analogous internal standards (3,6-F_2_-PBDE 99, PBDE 101) and proteins were denaturated with reagent grade alcohol. POP compounds were extracted with hexane from the aqueous matrix using a liquid-liquid extraction in the presence of a saturated ammonium sulfate solution for salting out. Thereafter, the extracts were evaporated to dryness before they were redissolved in 0.5 mL of hexane. These extracts were further purified on activated florisil columns and which were eluted with a mixture of dichloromethane:hexane (9 mL; 25:75) prior to GC-MS analysis.

The solvent was then evaporated, the residual taken up in 20 μL of hexane and analyzed for POP compounds on an Agilent 6890 Network or 7890A gas chromatograph (GC) coupled to an Agilent 5973 Network or 5975C mass spectrometer (MS) (Agilent Technologies; Mississauga, Ontario, Canada). The GC was fitted with an Agilent 60 m DB-XLB column (0.25 mm i.d., 0.25 μm film thickness) for detection by MS and with an Agilent Ultra-1 50 m (0.20 mm i.d., 0.33 μm film thickness) (Agilent Technologies; Mississauga, Ontario, Canada) column for the Electron Capture Detector (ECD). The carrier gas was helium and the injections were 3 μL in splitless mode. The mass spectrometer was operated in selected ion monitoring (SIM) mode, using negative chemical ionization (NCI) with methane (99.97 %) as the reagent gas. The ECD serves only to quantify PCB congeners 28 and 52 when the detection limits for these compounds were not achieved with the mass detector.

Concentrations were reported in units of micrograms per liter (μg/L) and the limits of detection (LOD) were between 0.005 and 0.3 μg/L for POPs. LODs were determined by first estimating concentrations of analytes yielding a signal to noise ratio of 3. Plasma samples spiked with analytes in concentrations ranging from 4 to 10 times the estimated LODs were analyzed (10 replicates) and standard deviations were multiplied by three to obtain the LODs. The method recoveries for each analyte varied from 68 to 90 %. The intra-day precision (repeatability) and the inter-day precision (reproducibility) of the method were respectively between 1.2 to 8.3 % and 2.8 to 13 %. Total cholesterol (TC), free cholesterol (FC), triglycerides (TG) and phospholipids (PL) levels were also measured in these samples by enzymatic methods combined with colorimetry (in g/L) at the laboratory of Centre Hospitalier de l’Université Laval (CHUL; Quebec, Quebec) and used to calculate the total lipid level as 1.677*(TC-FC) + FC + TG + PL [38].

The PFAS analysis was performed as described in the Canadian Health Measures Survey (CHMS) Cycle 1 report [2]. The LODs were between 0.1 to 0.3 μg/L for the compounds PFHxS, PFOA and PFOS. The intra-day precision varied between 5.4 and 6.3 % and the inter-day precision varied between 4.9 and 10 %.

The internal reference materials used to control the quality of the analyses were the certified reference material SRM-1958 (POPs and PFASs) from the National Institute of Standards and Technology (NIST; Gaithersburg, MD), the reference materials W-09-02 and W-10-06 (POPs) from the External Quality Assessment Scheme within the Arctic Monitoring and Assessment Program (AMAP) (Centre de toxicologie du Québec (CTQ), Institut National de Santé Publique du Québec (INSPQ), Québec, Canada) and some in-house reference materials for PFASs.

The overall quality and accuracy for both analytical methods was monitored by participation in the interlaboratory program AMAP External Quality Assessment Scheme (Centre de toxicologie du Québec (CTQ), Institut National de Santé Publique du Québec (INSPQ), Québec, Canada) as well as the German External Quality Assessment Scheme (G-EQUAS; Erlangen, Germany).

### Statistical analysis

As most contaminants had values below the limit of detection, censoring methods [39] were implemented for descriptive statistics, hypothesis tests and linear regressions. Such techniques are classified as either *parametric methods*, such as maximum likelihood (ML) estimation, or *nonparametric methods,* such as Kaplan-Meier (KM) and the generalized Wilcoxon test [39]. Total lipids were included in the models of all POPs other than the PFASs as a covariate.

Descriptive statistics including sample size, detection limit, percentage of observations below the limit of detection (LOD), minimum, median, 95th percentile, maximum, geometric mean, geometric standard error and associated 95 % confidence interval were reported for maternal and cord plasma concentrations of POPs, both adjusted and unadjusted for total lipids, with the exception of PFASs which were not lipid-adjusted. Note that the highest detection limit is presented in tables when several detection limits were noted.

For censoring methods, the geometric mean and 95 % confidence interval were calculated using the ML method and compared to the empirical median from the KM approach. The Greenwood estimate of variance was used for determination of Kaplan-Meier confidence intervals. Contaminants having the % < LOD of approximately 50 % were analyzed using the censored methods. Justification for including contaminants with up to 50 % censoring is provided in Helsel [39].

Demographic variables which were considered as potential predictors of contaminant concentrations, including parity, maternal age group (<25, 25–29, 30–34, 35+), smoking status (current, former, never), household income in Canadian dollars (≤$50,000, $50,001–$100,000, >$100,000), pre-pregnancy body mass index (BMI) based on self-reported pre-pregnancy weight and measured height and categorized as Underweight to Normal (BMI < 25); Overweight (25 < = BMI < 30); and Obese (BMI > =30), place of birth (foreign-born, Canadian born), fasting status (yes, no), maternal education (high school or less, some college or college degree, undergraduate degree or higher), sample collection year (2008–2011), consumption of: any kind of fish (never, 1, or 2+ times per week), bacon (never, 1, or 2+ times per week), hamburger (never, 1, 2, or >2 times per week), pork (never, 1, or 2+ times per week), poultry (never, 1, 2, or >2 times per week) and steak (never, 1, or 2+ times per week), use of non-stick cooking vessels (only for PFASs) and infant gender for cord plasma. Hypothesis tests were performed for contaminants with at least 50 % of observations above LOD [39] using the likelihood ratio test for parametric ML estimation and the nonparametric Wilcoxon rank-sum test. Bonferroni-adjusted confidence intervals were calculated and used to identify the different groups when overall tests were significant. To aid model selection, a test for log-normality with left-censored observations developed by Nysen et. al. [40] was used. If the assumption of normality failed, then nonparametric testing results were presented. Lipid adjusted models included total lipids of each sample as a covariate in the linear model with the potential predictor of interest.

In addition to performing the analysis described above, we also considered evaluation of infant cord plasma based on three demographic variables: infant gender, maternal smoking status and season of cord plasma collection. The maximum likelihood estimation (MLE) method was employed to account for left–censored repeated measures (twins and triplets), using nonlinear mixed models, analogous to Jin et. al. [41] and Thiebaut and Jacqmin-Gadda [42] which employed the NLMIXED procedure in SAS. Hypothesis testing was performed using likelihood ratio tests that followed a chi-square distribution.

Furthermore, prediction intervals were computed for geometric mean contaminant concentrations by the three demographic variables of interest, using Empirical Bayes estimates of the random effects [43]. Interpretations of these geometric means are similar to those computed for the previous models.

Linear associations among all unadjusted chemicals with >70 % detection were examined using the correlation coefficient. Spearman’s rho (ρ) was calculated since the contaminant concentrations were not normally distributed. Observations below the LOD were imputed by LOD/2. A heat map was plotted to represent Spearman correlation coefficient matrix.

For the 48 participants who had PFAS measurements in both the 1st and 3rd trimester we calculated intra-class correlation coefficients (ICCs). ICCs were calculated using a one-way random effects model (Proc Mixed) to estimate the between- and within-subject variability across both time points. The ICC measures the ratio of between-subject variance to total variance. It ranges from 0 to 1, with 0 meaning no within person reproducibility and 1 meaning perfect reproducibility.

Placental transfer was described by the calculation of the concentration ratios between paired cord and maternal samples for each compound on unadjusted and lipid adjusted concentrations (excluding the PFASs) using the formula R_cm_ = C_uc_ / C_m_ where C_uc_ is the umbilical cord concentration and C_m_ is the maternal concentration. Ratio calculations are only presented for those chemicals that had ≥10 pairs above the LOD [9].

Statistical analysis was performed using software packages SAS (Statistical Analysis System) Enterprise Guide 4.2 and R (R Core Development Team). For the censoring methods, functions from the R packages NADA and SURVIVAL were used for analysis. Unless otherwise indicated, a 5 % significance level (α = 0.05) was implemented throughout.

## Results

We found high detection rates in maternal blood for a number of chemicals but limited detection in cord blood. The PFASs were found to be highly reproducible in a small subset of samples that had PFASs measured in the 1st and 3rd trimester. Associations were found between and number of covariates, including parity, maternal age, income, and fish consumption in line with other studies. Foreign born participants appear to have higher levels of some chemicals. The maternal:cord plasma ratios are described and all fall below 1.

There were a total of 1983 pregnant women in the MIREC study [37]. The mean maternal age was 32.2 years and most of the women were in their 1st or 2nd pregnancy. The vast majority were Canadian born (81.3 %) and married (71.8 %) and close to 60 % had at least one university degree. Only 12 % were current smokers, while 27 % were former smokers. The majority were in the normal pre-pregnancy BMI ranges, however more than a third were overweight or obese. There were a total of 1959 live births in the study and 1517 cord plasma samples were collected. There were 1934 singletons, and 49 multiple births. The infants were 53 % male and 47 % female (See Table 1).

**Table 1:** Study population

Characteristic	Frequency	Percent
Maternal age group		
<25	139	7.01
25–29	459	23.15
30–34	709	35.75
35+	676	34.09
Smoking status		
Current	237	11.96
Former	542	27.36
Never	1202	60.68
Country of birth		
Foreign born	371	18.71
Canadian born	1612	81.29
Maternal education		
High school or less	175	8.83
Some college or college degree	572	28.87
Undergraduate degree or higher	1234	62.29
Marital status		
Married	1424	71.81
Other	559	28.19
Income		
≤$50,000	347	17.49
$50,001–$100,000	786	39.64
More than $100,000	757	38.17
Parity		
0	874	44.12
1	800	40.38
2+	307	15.5
Body mass index (BMI)		
Underweight to normal (BMI <25)	1164	63.36
Overweight (25 ≤ BMI <30)	404	21.99
Obese (BMI >30)	269	14.64
Fasting status		
No	1914	98
Yes	39	2
Season of sampling (1st trimester)		
Fall	575	29.41
Spring	448	22.92
Summer	464	23.73
Winter	468	23.94
Infant gender		
Male	1034	52.51
Female	926	47.03
Babies		
Singletons	1934	97.53
Twins	47	2.37
Triplets	2	0.10

Note: for income and infant gender, sum of percent is not 100, because there is a group (don’t know) was not included

Tables 2, 3, 4, 5, 6 and 7 report the overall summary statistics for each contaminant. In Table 2, the highest PFAS geometric mean (GM) in maternal plasma was PFOS (4.56 μg/L). PFOA, PFOS and PFHxS were detected in 23–64 % of the cord plasma. The GM of maternal PFOA was 4 times higher than that of cord plasma (1.65 vs 0.35 μg/L). There was a moderate and positive linear association among all the PFASs in maternal plasma (See Table 3). There was also a significant and positive correlation between maternal and cord plasma for PFOA.

**Table 2:** Descriptive statistics for PFASs in maternal and cord blood plasma (wet weight—μg/L)

Contaminant	Aliquot	N	LOD	% < LOD	Min	Median	95th Percentile	Max	KM Median	95 % CI		GM MLE	95 % CI	
PFHxS	Maternal	1940	0.3	5.31	ND	1	4.3	40	1	0.96	1.05	1.03	0.99	1.07
	Cord	1385	0.3	77.11	ND	ND	0.626	1.9	NA	NA	NA	NA	NA	NA
PFOA	Maternal	1940	0.1	0.15	ND	1.7	4.1	16	1.7	1.65	1.75	1.65	1.60	1.69
	Cord	1384	0.3	36.42	ND	0.39	1.1	5.6	0.39	0.37	0.41	0.35	0.33	0.36
PFOS	Maternal	1940	0.3	0.15	ND	4.6	11	36	4.6	4.47	4.73	4.56	4.45	4.68
	Cord	1385	0.3	52.27	ND	ND	1.5	5.8	NA	NA	NA	NA	NA	NA

*ND Non-detects (value below the limit of detection)*

*NA Non-applicable (KM median and GM MLE of contaminants with less than 50 % of observations detected were not calculated)*

**Table 3:** Correlations among PFOA, PFOS and PFHxS

Maternal plasma (1st trimester)	Spearman correlation	Maternal (1st trimester): Cord Plasma	Spearman correlation
PFOA: PFOS	0.56	PFOA: PFOA	0.67
PFOA: PFHxS	0.50		
PFOS: PFHxS	0.53		

Correlations between maternal and cord blood for PFOS and PFHxS not presented since more than 70 % of the observations were undetected

**Table 4:** Comparison of 1st trimester and 3rd trimester PFAS concentrations

	% below LOD	1st trimester GM	3rd trimester GM	ICC	Spearman correlation
PFOA	0	1.71	1.06	0.64 (0.46, 0.78)	0.90
PFOS	0	5.70	3.78	0.69 (0.53, 0.82)	0.91
PFHxS	5	0.98	0.70	0.83 (0.73, 0.90)	0.98

*N* = 48 paired samples measured in both the 1st and 3rd trimester of pregnancy

**Table 5:** Descriptive statistics for MIREC PCBs concentrations in maternal and cord blood plasma (lipid-adjusted—μg/kg)

Contaminant^a^	Aliquot	N	LOD	% < LOD	Min	Median	95th Percentile	Max	KM Median	95 % CI		GM	95 % CI	
Mono-ortho Dioxin-Like PCBs														
PCB105	Maternal	1935	0.01	95.71	ND	ND	ND	13.62	NA	NA	NA	NA	NA	NA
	Cord	1382	0.01	99.86	ND	ND	ND	100	NA	NA	NA	NA	NA	NA
PCB118	Maternal	1935	0.01	26.61	ND	2.33	6.78	37.93	2.30	2.22	2.37	2.36	2.29	2.43
	Cord	1382	0.01	98.34	ND	ND	ND	100	NA	NA	NA	NA	NA	NA
PCB156	Maternal	1935	0.01	79.07	ND	ND	3.52	17.46	NA	NA	NA	NA	NA	NA
	Cord	1380	0.01	99.71	ND	ND	ND	100.00	NA	NA	NA	NA	NA	NA
PCB167	Maternal	1935	0.01	98.40	ND	ND	ND	4.49	NA	NA	NA	NA	NA	NA
Non-dioxin-like PCBs														
PCB28	Maternal	1934	0.05	99.74	ND	ND	ND	260	NA	NA	NA	NA	NA	NA
PCB66	Maternal	1934	0.03	99.28	ND	ND	ND	28	NA	NA	NA	NA	NA	NA
PCB74	Maternal	1935	0.03	96.54	ND	ND	ND	18.4	NA	NA	NA	NA	NA	NA
PCB99	Maternal	1934	0.03	97.16	ND	ND	ND	12.93	NA	NA	NA	NA	NA	NA
PCB101	Maternal	1934	0.03	99.90	ND	ND	ND	12.07	NA	NA	NA	NA	NA	NA
PCB128	Maternal	1935	0.01	99.79	ND	ND	ND	5	NA	NA	NA	NA	NA	NA
PCB138	Maternal	1935	0.01	7.03	ND	4.07	14.77	71.67	4.07	3.91	4.23	4.21	4.08	4.35
	Cord	1382	0.01	95.08	ND	ND	ND	100	NA	NA	NA	NA	NA	NA
PCB146	Maternal	1935	0.01	85.43	ND	ND	2.93	16	NA	NA	NA	NA	NA	NA
	Cord	1382	0.01	99.86	ND	ND	ND	100	NA	NA	NA	NA	NA	NA
PCB153	Maternal	1935	0.01	1.29	ND	7	25.95	155	7	6.73	7.27	7.30	7.07	7.54
	Cord	1382	0.01	84.01	ND	ND	45.26	101.45	NA	NA	NA	NA	NA	NA
PCB170	Maternal	1935	0.01	46.82	ND	2	7.22	71.67	1.69	1.62	1.76	1.67	1.59	1.75
	Cord	1382	0.01	98.34	ND	ND	ND	100	NA	NA	NA	NA	NA	NA
PCB163	Maternal	1935	0.01	68.11	ND	ND	4.70	23.81	NA	NA	NA	NA	NA	NA
	Cord	1382	0.01	99.20	ND	ND	ND	100	NA	NA	NA	NA	NA	NA
PCB178	Maternal	1935	0.01	95.71	ND	ND	ND	9.02	NA	NA	NA	NA	NA	NA
PCB180	Maternal	1935	0.01	7.39	ND	4.72	19.70	183.33	4.72	4.53	4.91	4.88	4.70	5.06
	Cord	1382	0.01	93.63	ND	ND	41.67	100	NA	NA	NA	NA	NA	NA
PCB183	Maternal	1935	0.01	91.21	ND	ND	2.50	23.33	NA	NA	NA	NA	NA	NA
	Cord	1382	0.01	99.93	ND	ND	ND	100	NA	NA	NA	NA	NA	NA
PCB187	Maternal	1935	0.01	57.21	ND	ND	5.88	45	NA	NA	NA	NA	NA	NA
	Cord	1382	0.01	99.57	ND	ND	ND	100	NA	NA	NA	NA	NA	NA
PCB194	Maternal	1928	0.01	81.17	ND	ND	3.39	30	NA	NA	NA	NA	NA	NA
	Cord	1382	0.01	99.93	ND	ND	ND	100	NA	NA	NA	NA	NA	NA
PCB201	Maternal	1935	0.01	83.72	ND	ND	2.97	20	NA	NA	NA	NA	NA	NA
PCB203	Maternal	1935	0.01	89.35	ND	ND	2.56	14.17	NA	NA	NA	NA	NA	NA
PCB206	Maternal	1928	0.01	97.67	ND	ND	ND	6.61	NA	NA	NA	NA	NA	NA

*ND Due to a high percentage of non-detects, descriptive statistics were not reported*

*NA Non-applicable (KM median and GM MLE of contaminants with less than 50 % of observations detected were not calculated)*

^a^ *Contaminants PCB 52 was not detected in both maternal and cord blood samples. Contaminants PCB 101, PCB 128, PCB 167, PCB 178, PCB 201, PCB 203, PCB 206, PCB 28, PCB 66, PCB 74, PCB 99, were not detected in the cord blood*

**Table 6:** Descriptive statistics for MIREC Organochorine concentrations in maternal and cord blood plasma (lipid-adjusted—μg/kg)

Contaminant^a^	Aliquot	N	LOD	% < LOD	Min	Median	95th Percentile	Max	KM Median	95 % CI		GM	95 % CI	
Beta-HCH	Maternal	1898	0.01	31.88	ND	2.31	18.97	1108.11	2.24	2.15	2.32	2.32	2.19	2.46
	Cord	1379	0.01	93.55	ND	ND	47.62	740.74	NA	NA	NA	NA	NA	NA
Cis-nonachlor	Maternal	1934	0.005	88.31	ND	ND	1.25	3.91	NA	NA	NA	NA	NA	NA
DDE	Maternal	1935	0.09	1.03	ND	48.33	262.55	5306.12	48.33	46.68	49.99	56.02	54.02	58.08
	Cord	1382	0.09	87.48	ND	ND	452.50	1827.96	NA	NA	NA	NA	NA	NA
DDT	Maternal	1935	0.05	96.28	ND	ND	ND	175.44	NA	NA	NA	NA	NA	NA
	Cord	1381	0.05	99.71	ND	ND	ND	500	NA	NA	NA	NA	NA	NA
HCB	Maternal	1934	0.04	69.54	ND	ND	11.73	101.67	NA	NA	NA	NA	NA	NA
	Cord	1380	0.04	99.86	ND	ND	ND	400	NA	NA	NA	NA	NA	NA
Mirex	Maternal	1934	0.01	91.83	ND	ND	2.50	38.03	NA	NA	NA	NA	NA	NA
Oxychlordane	Maternal	1933	0.005	7.81	ND	2.09	4.60	17.5	2.09	2.02	2.15	2.01	1.96	2.06
	Cord	1378	0.005	97.97	ND	ND	ND	50	NA	NA	NA	NA	NA	NA
Parlar26	Maternal	1934	0.005	97.47	ND	ND	ND	3.65	NA	NA	NA	NA	NA	NA
Parlar50	Maternal	1934	0.005	87.28	ND	ND	1.22	5	NA	NA	NA	NA	NA	NA
Transnonachlor	Maternal	1934	0.01	15.87	ND	2.89	7.30	34.33	2.88	2.78	2.99	2.90	2.83	2.98
	Cord	1381	0.01	98.99	ND	ND	ND	100	NA	NA	NA	NA	NA	NA

*ND Due to a high percentage of non-detects, descriptive statistics were not reported*

*NA Non-applicable (KM median and GM MLE of contaminants with less than 50 % of observations detected were not calculated)*

^a^ *Aldrin, Alpha-chlordane, Gamma-Chlordane, and Gamma-HCH were not detected in both maternal and cord blood samples. Contaminants cis-nonachlor, Mirex, Parlar-26, and Parlar-50 were not detected in the cord blood*

**Table 7:** Descriptive statistics for MIREC PBB and PBDEs concentrations in maternal and cord blood plasma (lipid-adjusted—μg/kg)

Contaminant^a^	Aliquot	N	LOD	% < LOD	Min	Median	95th Percentile	Max	KM Median	95 % CI		GM	95 % CI	
PBB153	Maternal	1928	0.02	99.48	ND	ND	ND	13.62	NA	NA	NA	NA	NA	NA
PBDE100	Maternal	1927	0.02	78.46	ND	ND	10.16	327.27	NA	NA	NA	NA	NA	NA
	Cord	1379	0.02	99.06	ND	ND	ND	200	NA	NA	NA	NA	NA	NA
PBDE153	Maternal	1928	0.02	55.60	ND	ND	36.36	527.27	NA	NA	NA	NA	NA	NA
	Cord	1381	0.02	96.96	ND	ND	ND	221.05	NA	NA	NA	NA	NA	NA
PBDE28	Maternal	1928	0.03	99.07	ND	ND	ND	27.14	NA	NA	NA	NA	NA	NA
PBDE33	Maternal	1927	0.03	99.90	ND	ND	ND	8.57	NA	NA	NA	NA	NA	NA
PBDE47	Maternal	1928	0.03	34.28	ND	7.19	37.78	727.27	7.00	6.65	7.35	7.02	6.68	7.38
	Cord	1379	0.03	94.63	ND	ND	125	411.76	NA	NA	NA	NA	NA	NA
PBDE99	Maternal	1927	0.02	80.85	ND	ND	8.36	169.09	NA	NA	NA	NA	NA	NA
	Cord	1379	0.02	98.55	ND	ND	ND	1148.15	NA	NA	NA	NA	NA	NA

*ND Due to a high percentage of non-detects, descriptive statistics were not reported*

*NA Non-applicable (KM median and GM MLE of contaminants with less than 50 % of observations detected were not calculated)*

^a^ *PBDE 15, PBDE 17, and PBDE 25 were not detected in both maternal and cord blood samples. PBB 153, PBDE 28, PBDE 33, were not detected in the cord blood*

Table 4 describes the correlation between the paired 1st and 3rd trimester PFAS concentrations for a subset of women who had them measured at both time points. The 1st and 3rd trimester samples were highly correlated and had moderate to high reproducibility with ICCs ranging from 0.6 to 0.8. The 1st trimester samples were consistently higher than the 3rd trimester samples. The PCBs 118, 138, 153, and180 were the most highly detected PCBs (Table 5). DDE and oxychlordane were the most highly detected organochlorines (Table 6) while PBDE47 was the most highly detected flame retardant (See Table 7). Certain contaminants, namely aldrin, α-chlordane, γ-chlordane, γ-HCH, PBDE 15, PBDE 17, PBDE 25 and PCB 52 were not detected in either maternal or cord plasma samples. Cord plasma concentrations were very low or not detected for most of the PCBs and PBDEs.

In Table 8 we present the median cord:maternal plasma concentration ratios. The unadjusted median ratios (0.08–0.28) are much lower than the lipid adjusted (0.60–0.87).

**Table 8:** Cord-maternal plasma median concentration ratios (C_m_ = C_uc_ / C_m_)

	MIREC (plasma)			Vizcaino et al. 2014 [9] (serum)		Needham et al. 2011 [70] (serum)
Chemical	No. of pairs	Median	Median lipid adjusted	Median	Median lipid adjusted	Median
Beta-HCH	88	0.16	0.78	0.34	0.70	
DDE	168	0.15	0.74	0.34	0.68	
Oxychlordane	28	0.21	0.87			
PBDE100	12	0.08	0.60			
PBDE153	40	0.10	0.48	0.31	0.58	0.34^a^
PBDE47	67	0.15	0.84	0.58	0.90	
PCB118	23	0.21	0.80	0.45	0.98	
PCB138	67	0.16	0.70	0.39	0.81	
PCB153	215	0.14	0.72	0.37	0.75	
PCB163	11	0.23	0.80			
PCB170	23	0.14	0.71			
PCB180	86	0.13	0.67	0.28	0.57	
Transnonachlor	14	0.19	0.79			
PFHxS	315	0.23				0.74
PFOA	865	0.28				0.72
PFOS	648	0.14				0.34

Note: only chemicals with >10 pairs shown (where both the maternal and cord blood concentrations were above the LOD)

^a^mean

Note that these ratios are only presented for pairs of maternal and cord plasma samples that are both above the LOD. The highest number of pairs was for PFOA (*n* = 865) while the highest median ratios (lipid adjusted) were for oxychlordane and PBDE47.

**Fig. 1:**
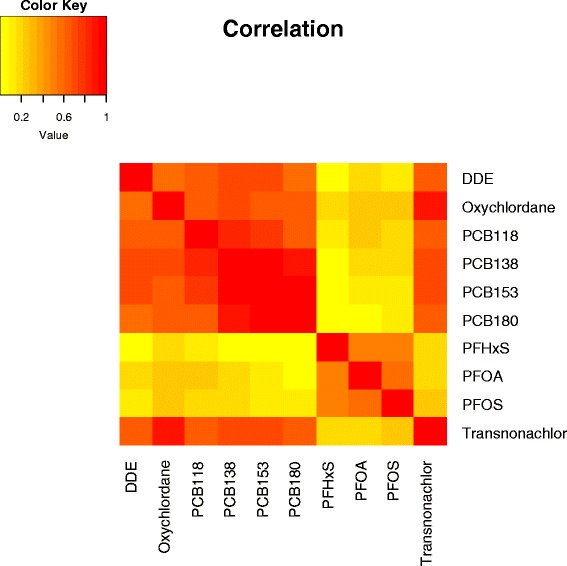
Correlation Heat Map (Maternal Plasma). *All chemicals with >70 % detection*

The median number of POPs detected in maternal plasma above the LOD was 14 (mean 15) with a maximum of 33. In cord plasma, very few chemicals were detected with a mean and median of 2 chemicals per cord plasma sample and a maximum of 19 chemicals. Figure 1 describes the correlation across chemicals in maternal plasma for those chemicals with >70 % above the LOD. The organochorine pesticides and PCBs showed moderate to high correlation with each other but low correlation with the PFASs. The PFASs were moderately correlated with each other.

Demographic variables were explored for contaminants having at least 50 % of the observations above the LOD and included: β-HCH, DDE, oxychlordane, PBDE153, PBDE47, PCB118, PCB138, PCB153, PCB180, trans-nonachlor, PFHxS, PFOA, and PFOS. Sufficient detection permitted consideration of PCB 170 levels by parity and place of birth only (See Table 9 for summary and Additional file 1 for further details).

**Table 9:** Hypothesis testing summary

	Beta-HCH	DDE	Oxychlordane	PBDE47	PCB118	PCB138	PCB153	PCB180	Transnonachlor	PFHxS	PFOA	PFOS
Increasing parity	-	-	-	NS	-	-	-	NS*	-	-	-	-
Increasing maternal age	NA	+	+	NS*	NA	+	+	+	NA	-	-	NS*
Current smokers	-	-	-	NS*	-	-	+	-	-	NS*	-	NS*
High household income > $100,000 CDN	+	NS*	+	NS*	+	+	+	+	+	NS*	+	+
High pre-pregnancy BMI	+	-	-	+	-	-	-	-	-	NS*	NS	NS
Foreign born	+	+	+	-	+	+	+	+	+	-	NS	-
Fasting at time of sample	NS	-	-	NS	NS	-	NS	NS	-	NS	NS	NS
Lower maternal education	-	-	-	NS*	-	-	-	-	-	NS	NS*	-
Sampling year (2011 vs 2008)	NA	-	-	-	-	-	-	-	-	NS	-	-
Fish consumption	+	+	+	NS*	+	+	+	+	+	NS	NS	NS
Bacon consumption	NA*	-	NS	NA*	NS*	NS	NS	NS	NS	NS	NS	NS
Hamburger consumption	-	-	NS*	NS	-	-	-	-	NS*	NS	NS	NS
Pork consumption	NS	NS	NS	NS	NS	NS	NS	NS	NS	NS	NS	NS
Poultry consumption	NS	NS	NS*	NS	NS*	NS	NS*	NS*	NS	NS	NS	NS
Steak consumption	NS	NS	NS	NS	NS	NS	NS	NS	NS	NS	NS	NS
Cook with non-stick cooking vessels										NS	+	NS

NA = not sufficient detection (>70 % below LOD) in categories for analysis

NA* = significant interaction with chemical and lipids. See Additional file 1 for details

NS = No statistically significant differences between groups

NS* = overall a significant effect was seen for this characteristic (i.e. parity, maternal age, smoking status, income, BMI, education level) but the test could not identify with sufficient confidence which pairs of means differ. The Bonferroni-adjusted confidence intervals are a very conservative method

- means a significant negative association (e.g. as parity increases DDE concentrations decrease)

+ means a significant positive association (e.g. as maternal age increases DDE concentrations increase)

Significant differences in contaminant concentrations were found by parity group for all contaminants considered except PBDE 47, with and without lipid adjustment. Generally, as the parity increases, the contaminant concentrations tended to decrease. We saw that contaminant levels were significantly higher in the older participants for the contaminants DDE, oxychlordane, PCB 138, PCB 153, and PCB180. However for two of the PFASs, the age group 35+ had significantly lower concentrations of PFHxS and PFOA than the younger age groups.

There were significant differences by smoking status for all contaminants considered with and without lipid adjustment except for PFOS. In general, non-smokers and former smokers had higher contaminant concentrations compared to current smokers. Only PBDE153 had higher concentrations in current smokers than non-smokers.

For household income we saw significant differences with and without lipid adjustment. Generally, as household income increased, the contaminant concentrations tended to increase in many cases, hence mothers with a household income of more than $100,000 CDN had significantly higher contaminant residue levels.

The majority of the lipid adjusted contaminants had significantly higher concentrations in underweight/normal weight mothers compared to obese mothers. However, there was an exception for β-HCH and PBDE-47, where significantly higher concentrations were seen for obese women in most cases.

Contaminant concentrations were significantly higher (unadjusted and lipid adjusted) in foreign-born mothers compared to Canadian-born mothers for all contaminants except PBDE 47 and the PFASs (PFHxS and PFOS), where higher concentrations were seen in Canadian-born mothers.

Fasting status demonstrated significant differences for DDE, oxychlordane, PCB 138 and trans-nonachlor with and without lipid adjustment. Contaminant residue levels were higher among those mothers who did not fast. For the other chemicals, no significant difference at the 5 % significance level was found with lipid adjustment.

There were significant differences by education level for nearly all contaminants. Generally, mothers with higher education had higher contaminant concentrations. Sampling year showed a significant effect for nearly all POPs. Further to this, women who participated in the study in 2011 had significantly lower concentrations of nearly all POPs compared to women who participated in 2008.

Fish consumers had significantly higher levels of nearly all POPs excluding the PFASs. Bacon, pork, poultry and steak consumption showed little effect on contaminant concentrations however participants who ate hamburger 2 or more times per week showed significantly lower levels of the OC pesticides β-HCH, DDE, and the PCBs (PCBs 118, 138, 153, 180). Only PFOA levels were higher for participants who cooked with non-stick cooking vessels. However no association was observed if they microwaved with non-stick cooking vessels.

No significant differences were detected when grouping by infant gender, maternal smoking status or season of collection for PFOA in cord plasma (See Additional file 1).

## Discussion

In maternal plasma, there was >90 % detection for DDE, oxychlordane, PCB 138, PCB 153, and the PFASs. Cord plasma had much lower detection rates with low or very limited detection for most PCBs and PBDEs. The highest detection rate in cord plasma was for the PFASs at 23 (PFHxS), 48 (PFOS) and 64 (PFOS) percent. In a small subsample we found maternal plasma PFASs concentrations to be highly correlated and to have good reproducibility between the 1st and 3rd trimester (ICCs 064-0.83). The cord:maternal plasma concentration ratios ranged from 0.14 (PFOS) to 0.87 (oxychlordane, lipid adjusted). Similar to other studies, we found parity, maternal age, income, education, smoking status, pre-pregnancy BMI and fish consumption to be significant predictors for most chemicals. Those participants who were foreign-born had significantly higher concentrations of organochlorinated pesticides and PCBs.

In comparison to other national surveys, the participants of the MIREC cohort had lower geometric mean concentrations of DDE. MIREC’s DDE concentrations were lower than those measured in females from the population-based Canadian Health Measures Survey (CHMS) [2] as well as females [4] and pregnant women [44] from the U.S. National Health And Nutrition Examination Survey (NHANES) study. Maternal trans-nonachlor and oxychlordane concentrations appear to be similar in MIREC to those found in the CHMS [2] and another Canadian study [45], and lower than that found in NHANES. The predominant PCB congeners were 138, 153, and 180 in the MIREC maternal plasma samples. This is consistent with other biomonitoring surveys in Canada [46]. Among the dioxin-like PCBs measured in MIREC, only PCB 118 was consistently detected in maternal plasma. PCB 118 concentrations were similar to those found in the CHMS [2] however, lower than other similar studies [4, 34, 45, 47, 72]. In cord plasma PCBs were rarely detected.

In the MIREC study cord plasma, the PFASs were detected more frequently than any other POPs. PFOA and PFOS had detection rates of 64 and 48 %, which is in contrast to the OC pesticides and PCBs that had only a 7–15 % detection rate. The 1st and 3rd trimester PFASs were found to be strongly correlated and moderately to strongly reproducible, with 1st trimester levels consistently higher, and ICCs ranging from 0.64 (PFOA) to 0.83 (PFHxS). Fei et al. [48] also found a high degree of correlation between 1st and 2nd trimester concentration of PFOA (*r* = 0.88) and PFOS (*r* = 0.87), with mean levels being higher in the 1st trimester. The MIREC maternal PFASs concentrations were similar to those found in females aged 20–39 in the population based study CHMS and another Canadian pregnancy cohort study based in Vancouver (CHiRP) [49] both conducted around the same time as MIREC. The MIREC PFOA and PFOS maternal concentrations are lower than those observed in earlier studies from the U.S. [3, 44], Norway [50], Denmark [51] and the Family study [52] from Hamilton, Ontario Canada. Cord plasma PFOA was significantly correlated with maternal plasma concentrations (*p* < 0.0001), as seen in other studies [8]. The MIREC PFOA cord plasma concentrations are also lower than those seen in other Canadian, and international studies, but similar to those reported in the Norwegian Birth Cohort (MoBa) [53].

PBDE 47 was the most frequently detected PBDE in the MIREC study. However, the MIREC PBDE 47 concentrations are notably lower than those found in earlier studies of pregnant women [44] and females [4] from the U.S. NHANES study and the Family study from Hamilton, Ontario [54]. Concentrations are similar to that found in the CHMS [2], conducted at the same time as MIREC, and the Canadian centres of an earlier Trinational study [45].

Parity, maternal age, income, education, smoking status, pre-pregnancy BMI, year of collection and fish consumption were found to be significant predictors for most chemicals. Foreign Born participants had higher levels of PCBs and organochlorinated pesticides.

Generally, as parity increases contaminant concentrations tended to decrease. The organochlorinated concentrations were significantly higher in older age groups, except the PFASs where both PFHxS and PFOA had lower concentrations in older mothers. Fei et al. [48, 51] also found declining PFASs concentrations with increasing age. In MIREC, obese participants had lower concentrations of DDE, oxychlordane, and the PCBs, and higher concentrations of β-HCH, and PBDE 47 compared to other lower weight categories. Other studies have shown lower PCB concentrations with increasing BMI [55] or no association with BMI [56].

Most contaminants were found to be higher in non-smokers and former smokers than current smokers, except for PFOS. This is in contrast to the findings in other studies which have shown a positive association with smoking and POP concentrations [57–61] or show no difference by smoking status for DDE [55], PCBs [55], and PFASs [50]. Our finding may be due to unmeasured confounders given we looked at the characteristics univariately. However results from Ayotte et al. [62] suggested that smoking induces liver CYP1A2 activity, which alters porphyrin metabolism and increases the biotransformation of mono-ortho PCBs.

OCs are highly lipophilic and partition among various tissues depending on their lipid content. Phillips et al. [63] showed increases of 20 % in PCB, HCB and DDE concentrations in 20 adults following a meal. However, they found no difference when concentrations were lipid adjusted. In the MIREC study, the few participants who fasted (*n* = 39) had significantly lower OC concentrations (DDE, oxychlordane, PCB 138 and trans-nonachlor) with and without lipid adjustment, suggesting that lipid adjustment alone may be insufficient to account for postprandial changes in these contaminant concentrations in pregnant women and other confounding measures must be considered.

Foreign-born participants had significantly higher concentrations of organochlorinated pesticides and PCBs. Foreign born participants were mainly from Europe (35 %) and Asia (19 %), followed by the Caribbean, South America and the U.S (all 9 %), Africa (8 %), Middle East (7 %) and Oceania (1 %). A Trinational Biomonitoring study [45] also showed consistently higher concentrations in Canadian immigrants than in Canadian born participants. Curren et al. [64] found that foreign-born Canadians had higher concentrations of DDE and β-HCH compared to Canadian-born and Inuit mothers. These exposures may originate from imported foods or exposures that occurred outside of Canada [65] or it may be due to different cultural habits and lifestyles related to their country of origin.

Both fish and hamburger consumption showed significant effects on contaminant concentrations albeit in different directions. Fish consumers had significantly higher levels of most POPs (excluding the PFASs). Fish consumption is known to affect the levels of POPs [66–68]. Hamburger consumption had a significant negative effect on POP levels. This finding is not supported in the literature as several studies have reported POPs in meat [69] and may reflect the fact that these participants are less likely to eat fish, where we saw a strong association with POPs. Among the women who reported eating hamburger 2 or more times per week, 60 % reported never eating fish.

We found that the year of the sample collection had a significant effect on POP concentrations. Lower levels were seen in 2011 compared to 2008 for nearly all POPs except PFHxS. This could reflect the fact that POP levels are decreasing over time as a result of bans. It may also reflect a difference in recruitment methods over time (e.g. older moms) and should be noted as an important consideration in cohort studies collecting data over a number of years.

The lipid adjusted cord:maternal serum concentration ratios for the OC pesticides, PBDEs and PCBs are all less than 1 and similar to a recent study from Spain [9] which analyzed samples from 308 mother-cord plasma pairs between 2004-08 (See Table 8). For the PFASs, PFOA had the highest median ratio (0.28) but is much lower than in a study of 15 women from a Faroe Islands fishing community where elevated exposures to marine contaminants occur [70]. It has been suggested that pollutant properties may affect the transport of pollutants from the mother to the fetus however Vizcaino et al. [9] did not show any correlation with pollutant properties such as molecular weight, molar volume, number of halogen substituents or log octanol water partition coefficient (K_ow_) and concentration ratios. Further exploration into the predictors of the maternal:fetal concentration ratio is warranted.

An examination of the chemicals measured in the serum of the pregnant women shows that most women had multiple PCBs, PBDEs and PFASs detected in their plasma. This result is supported by the work by Woodruff et al. [44] who showed that each pregnant woman in the NHANES study had at least 2 OC pesticides, one PBDE, two PFASs and four phthalates. Traditionally research has focused on single chemicals in the exposure assessment; however, there is growing recognition of the need to better assess the risk of mixtures and to understand the cumulative effects of multiple exposures and stressors [71].

One limitation and possible reason for lower concentrations in pregnant women compared to non-pregnant females in the CHMS is that we did not adjust for albumin. Albumin measurements can be used as a surrogate for plasma volume expansion in pregnancy. This plasma volume expansion may dilute environmental chemical concentrations in the plasma [44, 72]. Woodruff et al. [44] found that adjusting for albumin generally increased the GM estimates of persistent compounds (e.g. DDE) but not for non-persistent compounds. However, they suggest that the role of albumin as a transport protein during pregnancy requires further investigation. MIREC was also based on a convenience sample and is therefore not population-based and representative. Our results may also be biased towards women with higher age at delivery, education and income [37].

## Conclusions

Multiple chemicals were detectable in the serum of the pregnant women in our study cohort, however the concentrations were much lower than those seen in other studies. We found parity, maternal age, income, education, smoking status, pre-pregnancy BMI, fish consumption and year of collection to be significant predictors for most chemicals. Foreign-born participants had significantly higher concentrations of OCs and PCBs. In maternal plasma the highest detection rates were seen for the PFOS, PFOA, DDE, and PCB153. However in cord plasma, PFOA had the highest detection rate. In a small subset of participants we found the PFASs maternal plasma concentrations to be highly correlated and to have moderate to high reproducibility between the 1st and 3rd trimester.

## Additional file

Additional file 1: Results of Statistical Hypothesis Testing—MIREC Persistent Organic Pollutants Analysis-Using censoring methods. Table S1 Results for MIREC persistent organic pollutants in maternal blood by Parity (μg/L). Table S2 Results for MIREC persistent organic pollutants in maternal blood by maternal age (μg/L). Table S3 Results for MIREC persistent organic pollutants in maternal blood by smoking status (μg/L). Table S4 Results for MIREC persistent organic pollutants in maternal blood by household income (μg/L). Table S5 Results for MIREC persistent organic pollutants in maternal blood by pre-BMI (μg/L). Table S6 Results for MIREC persistent organic pollutants in maternal blood by place of birth (μg/L). Table S7 Results for MIREC persistent organic pollutants in maternal blood by fasting (μg/L). Table S8 Results for MIREC persistent organic pollutants in maternal blood by maternal education (μg/L). Table S9 Results for MIREC persistent organic pollutants in maternal blood by whether using non-stick cooking vessels. Table S10 Results for MIREC persistent organic pollutants in maternal blood by whether using non-stick cooking vessels in the microwave. Table S11 Results for MIREC persistent organic pollutants in maternal blood by year of collection. Table S12 Results for MIREC persistent organic pollutants in maternal blood by intake of bacon. Table S13 Results for MIREC persistent organic pollutants in maternal blood by intake of fish. Table S14 Results for MIREC persistent organic pollutants in maternal blood by intake of Hamburger. Table S15 Results for MIREC persistent organic pollutants in maternal blood by intake of pork. Table S16 Results for MIREC persistent organic pollutants in maternal blood by intake of poultry. Table S17 Results for MIREC persistent organic pollutants in maternal blood by intake of steak. Table S18 Comparison of demographic groups when the INTERACTION between BMI and total lipid was significant. Table S19 Comparison of demographic groups when the INTERACTION was significant between year of collection and total lipid. Table S20 Comparison of demographic groups when the INTERACTION was significant between intake of bacon and total lipid. Table S21 Results for MIREC persistent organic pollutants in cord blood by infant gender (μg/L). Table S22 Results for MIREC persistent organic pollutants in cord blood by season of collection (μg/L). Table S23 Results for MIREC persistent organic pollutants in cord blood by smoking status of mother (µg/L). (DOCX 207 kb)

## Abbreviations

BMI: body mass index
CHMS: Canadian Health Measures Survey
CTQ: Centre de Toxicologie du Québec
DDE: dichlorodiphenyldichloroethylene
DDT: p,p’-Dichlorodiphenyltrichloroethane
HCB: hexachlorobenzene
INSPQ: Institute National de Santé Publique
KM: Kaplan-Meier
LOD: limit of detection
MIREC: maternal-infant research on environmental chemicals
ML: maximum likelihood
MLE: maximum likelihood estimation
OCs: organochlorines
PBDEs: polybrominated diphenyl ethers
PCB: polychlorinated biphenyls
PFASs: perfluoroalkyl substances
PFASs: perfluoroalkyl substances
PFHxS: perfluorohexane sulfonate
PFOA: perfluorooctanoic acid
PFOS: perfluoroctane sulfonate
POPs: persistent organic pollutants
Tox 26: toxaphene parlar 26
Tox50: toxaphene parlar 50
β-HCH: β-Hexachlorocylcohexane
γ-HCH: γ-Hexachlorocylcohexane
