# Supplementary material for: Concentrations of persistent organic pollutants in maternal and cord blood from the maternal-infant research on environmental chemicals (MIREC) cohort study
Source: Environ Health. 2016 May 4;15:59. doi: 10.1186/s12940-016-0143-y (PMC4855498; doi:10.1186/s12940-016-0143-y)
Supplement: Additional file 1: — Results of Statistical Hypothesis Testing—MIREC Persistent Organic Pollutants Analysis-Using censoring methods. Table S1 Results for MIREC persistent organic pollutants in maternal blood by Parity (μg/L). Table S2 Results for MIREC persistent organic pollutants in maternal blood by maternal age (μg/L). Table S3 Results for MIREC persistent organic pollutants in maternal blood by smoking status (μg/L). Table S4 Results for MIREC persistent organic pollutants in maternal blood by household income (μg/L). Table S5 Results for MIREC persistent organic pollutants in maternal blood by pre-BMI (μg/L). Table S6 Results for MIREC persistent organic pollutants in maternal blood by place of birth (μg/L). Table S7 Results for MIREC persistent organic pollutants in maternal blood by fasting (μg/L). Table S8 Results for MIREC persistent organic pollutants in maternal blood by maternal education (μg/L). Table S9 Results for MIREC persistent organic pollutants in maternal blood by whether using non-stick cooking vessels. Table S10 Results for MIREC persistent organic pollutants in maternal blood by whether using non-stick cooking vessels in the microwave. Table S11 Results for MIREC persistent organic pollutants in maternal blood by year of collection. Table S12 Results for MIREC persistent organic pollutants in maternal blood by intake of bacon. Table S13 Results for MIREC persistent organic pollutants in maternal blood by intake of fish. Table S14 Results for MIREC persistent organic pollutants in maternal blood by intake of Hamburger. Table S15 Results for MIREC persistent organic pollutants in maternal blood by intake of pork. Table S16 Results for MIREC persistent organic pollutants in maternal blood by intake of poultry. Table S17 Results for MIREC persistent organic pollutants in maternal blood by intake of steak. Table S18 Comparison of demographic groups when the INTERACTION between BMI and total lipid was significant. Table S19 Comparison of demographic groups when the IN [file 12940_2016_143_MOESM1_ESM.docx]

**Results of Statistical Hypothesis Testing – MIREC Persistent Organic Pollutants Analysis-Using censoring methods**

[Table S1: Results for MIREC persistent organic pollutants in maternal blood by Parity (µg/L) 3](#_Toc442863607)

[Table S2: Results for MIREC persistent organic pollutants in maternal blood by maternal age (µg/L) 5](#_Toc442863608)

[Table S3: Results for MIREC persistent organic pollutants in maternal blood by smoking status (µg/L) 8](#_Toc442863609)

[Table S4: Results for MIREC persistent organic pollutants in maternal blood by household income (µg/L) 10](#_Toc442863610)

[Table S5: Results for MIREC persistent organic pollutants in maternal blood by pre-BMI (µg/L) 12](#_Toc442863611)

[Table S6: Results for MIREC persistent organic pollutants in maternal blood by place of birth (µg/L) 15](#_Toc442863612)

[Table S7: Results for MIREC persistent organic pollutants in maternal blood by fasting (µg/L) 17](#_Toc442863613)

[Table S8: Results for MIREC persistent organic pollutants in maternal blood by maternal education (µg/L) 19](#_Toc442863614)

[Table S9: Results for MIREC persistent organic pollutants in maternal blood by whether using non-stick cooking vessels 22](#_Toc442863615)

[Table S10: Results for MIREC persistent organic pollutants in maternal blood by whether using non-stick cooking vessels in the microwave 22](#_Toc442863616)

[Table S11: Results for MIREC persistent organic pollutants in maternal blood by year of collection 23](#_Toc442863617)

[Table S12: Results for MIREC persistent organic pollutants in maternal blood by intake of bacon 26](#_Toc442863618)

[Table S13: Results for MIREC persistent organic pollutants in maternal blood by intake of fish 28](#_Toc442863619)

[Table S14: Results for MIREC persistent organic pollutants in maternal blood by intake of Hamburger 29](#_Toc442863620)

[Table S15: Results for MIREC persistent organic pollutants in maternal blood by intake of pork 32](#_Toc442863621)

[Table S16: Results for MIREC persistent organic pollutants in maternal blood by intake of poultry 34](#_Toc442863622)

[Table S17: Results for MIREC persistent organic pollutants in maternal blood by intake of steak 37](#_Toc442863623)

[Table S18: Comparison of demographic groups when the INTERACTION between BMI and total lipid was significant 39](#_Toc442863624)

[Table S19: Comparison of demographic groups when the INTERACTION was significant between year of collection and total lipid 39](#_Toc442863625)

[Table S20: Comparison of demographic groups when the INTERACTION was significant between intake of bacon and total lipid 40](#_Toc442863626)

[Table S21: Results for MIREC persistent organic pollutants in cord blood by infant gender (µg/L) 41](#_Toc442863627)

[Table S22: Results for MIREC persistent organic pollutants in cord blood by season of collection (µg/L) 41](#_Toc442863628)

Groups having the same letter indicate levels that are statistically similar, whereas groups with different letters represent levels that are statistically different. Confidence intervals reported are corrected for multiple pair-wise comparisons (Bonferroni-adjusted). Total lipid was significant for all contaminants. Hence, a strong linear relationship exists between total lipid and POPs studied.

# Table S1: Results for MIREC persistent organic pollutants in maternal blood by Parity (µg/L)

| **Contaminant** | **Group** | **N** | **%<LOD** | **Unadjusted** | | | **Lipid as a covariate** | | | |
| --- | --- | --- | --- | --- | --- | --- | --- | --- | --- | --- |
|  |  |  |  | **P-Value** | **GM* (95% CI)** | | **P-Value for lipids** | **P-Value** | **GM* (95% CI)** | |
| Beta-HCH | 0 | 835 | 24.31 | <.0001 | A | 0.0173 (0.0155, 0.0193) | <.0001 | <.0001 | A | 0.0175 (0.0158, 0.0194) |
|  | 1 | 770 | 34.68 |  | B | 0.0133 (0.0119, 0.0148) |  |  | B | 0.0130 (0.0116, 0.0145) |
|  | 2+ | 291 | 46.05 |  | B | 0.0100 (0.0083, 0.0122) |  |  | C | 0.0097 (0.0080, 0.0116) |
| DDE | 0 | 852 | 0.35 | <.0001 | A | 0.3897 (0.3639, 0.4174) | <.0001 | <.0001 | A | 0.3902 (0.3655, 0.4166) |
|  | 1 | 780 | 1.03 |  | B | 0.3264 (0.3055, 0.3487) |  |  | B | 0.3267 (0.3051, 0.3499) |
|  | 2+ | 301 | 2.99 |  | C | 0.2729 (0.2440, 0.3053) |  |  | C | 0.2710 (0.2427, 0.3027) |
| Oxychlordane | 0 | 852 | 5.75 | <.0001 | A | 0.0137 (0.0131, 0.0143) | <.0001 | <.0001 | A | 0.0137 (0.0131, 0.0143) |
|  | 1 | 778 | 7.71 |  | B | 0.0118 (0.0112, 0.0124) |  |  | B | 0.0118 (0.0113, 0.0124) |
|  | 2+ | 301 | 13.95 |  | C | 0.0100 (0.0092, 0.0109) |  |  | C | 0.0099 (0.0092, 0.0107) |
| PBDE47 | 0 | 850 | 33.18 | 0.2356 |  | 0.0447 (0.0408, 0.0489) | <.0001 | 0.2598 |  | 0.0445 (0.0407, 0.0487) |
|  | 1 | 778 | 36.38 |  |  | 0.0408 (0.0369, 0.0451) |  |  |  | 0.0410 (0.0373, 0.0451) |
|  | 2+ | 298 | 31.54 |  |  | 0.0451 (0.0385, 0.0528) |  |  |  | 0.0448 (0.0386, 0.0521) |
| Arochlor1260 | 0 | 852 | 2.70 | <.0001 | A | 0.3898 (0.3668, 0.4142) | <.0001 | <.0001 | A | 0.3903 (0.3682, 0.4137) |
|  | 1 | 780 | 2.44 |  | AB | 0.3667 (0.3454, 0.3893) |  |  | AB | 0.3670 (0.3454, 0.3901) |
|  | 2+ | 301 | 3.32 |  | B | 0.3141 (0.2841, 0.3473) |  |  | B | 0.3118 (0.2827, 0.3440) |
| PCB118 | 0 | 852 | 20.89 | <.0001 | A | 0.0160 (0.0152, 0.0169) | <.0001 | <.0001 | A | 0.0160 (0.0152, 0.0169) |
|  | 1 | 780 | 28.46 |  | B | 0.0140 (0.0132, 0.0148) |  |  | B | 0.0140 (0.0132, 0.0148) |
|  | 2+ | 301 | 37.87 |  | C | 0.0119 (0.0108, 0.0132) |  |  | C | 0.0119 (0.0109, 0.0130) |
| PCB138 | 0 | 852 | 6.46 | <.0001 | A | 0.0275 (0.0260, 0.0292) | <.0001 | <.0001 | A | 0.0276 (0.0261, 0.0292) |
|  | 1 | 780 | 6.41 |  | AB | 0.0257 (0.0243, 0.0273) |  |  | AB | 0.0257 (0.0243, 0.0273) |
|  | 2+ | 301 | 9.97 |  | B | 0.0218 (0.0197, 0.0241) |  |  | B | 0.0216 (0.0197, 0.0238) |
| PCB153 | 0 | 852 | 1.29 | 0.0002 | A | 0.0473 (0.0444, 0.0503) | <.0001 | 0.0001 | A | 0.0473 (0.0446, 0.0502) |
|  | 1 | 780 | 1.15 |  | AB | 0.0447 (0.0420, 0.0475) |  |  | AB | 0.0447 (0.0420, 0.0476) |
|  | 2+ | 301 | 1.66 |  | B | 0.0385 (0.0347, 0.0426) |  |  | B | 0.0382 (0.0345, 0.0422) |
| PCB170++ | 0 | 852 | 45.31 | 0.0056 | A | 0.0108 (0.0099, 0.0118) | <.0001 | 0.0038 | A | 0.0108 (0.0100, 0.0118) |
|  | 1 | 780 | 45.64 |  | A | 0.0105 (0.0097, 0.0115) |  |  | AB | 0.0104 (0.0096, 0.0113) |
|  | 2+ | 301 | 54.15 |  | A | 0.0085 (0.0072, 0.0101) |  |  | B | 0.0087 (0.0076, 0.0100) |
| PCB180 | 0 | 852 | 8.10 | 0.0164** | AB | 0.029 (0.0268, 0.0312) | <.0001 | 0.0418 | A | 0.0304 (0.0284, 0.0325) |
|  | 1 | 780 | 6.79 |  | A | 0.030 (0.0278, 0.0322) |  |  | A | 0.0306 (0.0286, 0.0328) |
|  | 2+ | 301 | 6.98 |  | B | 0.025 (0.0223, 0.0277) |  |  | A | 0.0268 (0.0240, 0.0300) |
| Transnonachlor | 0 | 852 | 13.73 | <.0001 | A | 0.0193 (0.0184, 0.0203) | <.0001 | <.0001 | A | 0.0194 (0.0185, 0.0204) |
|  | 1 | 780 | 15.64 |  | B | 0.0175 (0.0166, 0.0184) |  |  | B | 0.0175 (0.0166, 0.0184) |
|  | 2+ | 300 | 22.33 |  | C | 0.0149 (0.0137, 0.0161) |  |  | C | 0.0147 (0.0135, 0.0159) |
| PFHxS | 0 | 856 | 3.15 | <.0001 | A | 1.2609 (1.1826, 1.3444) |  |  |  |  |
|  | 1 | 780 | 3.46 |  | B | 0.9433 (0.8859, 1.0044) |  |  |  |  |
|  | 2+ | 302 | 8.61 |  | C | 0.7270 (0.6493, 0.8140) |  |  |  |  |
| PFOA | 0 | 856 | 0.23 | <.0001 | A | 2.2182 (2.1322, 2.3075) |  |  |  |  |
|  | 1 | 780 | 0.13 |  | B | 1.3538 (1.2911, 1.4196) |  |  |  |  |
|  | 2+ | 302 | 0.00 |  | C | 1.1743 (1.0891, 1.2662) |  |  |  |  |
| PFOS | 0 | 856 | 0.23 | <.0001 | A | 5.3343 (5.0867, 5.5938) |  |  |  |  |
|  | 1 | 780 | 0.00 |  | B | 4.2555 (4.0648, 4.4551) |  |  |  |  |
|  | 2+ | 302 | 0.33 |  | C | 3.5097 (3.2478, 3.7927) |  |  |  |  |

^*^ 95% CI: the 95% confidence intervals for the geometric mean were corrected for multiple comparisons using Bonferroni correction

^**^ The analysis is based on the non-parametric Wilcoxon test and Kaplan-Meier estimates of the median. Confidence intervals were calculated using the Greenwood variance estimate.

++ Caution should be taken since one group has more than 50% observations undetected.

# Table S2: Results for MIREC persistent organic pollutants in maternal blood by maternal age (µg/L)

| **Contaminant** | **Group** | **N** | **%<LOD** | **Unadjusted** | | | **lipid as a covariate** | | | |
| --- | --- | --- | --- | --- | --- | --- | --- | --- | --- | --- |
|  |  |  |  | **P-Value** | **GM* (95% CI)** | | **P-Value for lipids** | **P-Value** | **GM* (95% CI)** | |
| DDE | <25 | 138 | 7.25 | <.0001 | A | 0.1962 (0.1696,0.2270) | <.0001 | <.0001 | A | 0.2007 (0.1680,0.2396) |
|  | 25-29 | 448 | 0.45 |  | B | 0.2943 (0.2684,0.3227) |  |  | B | 0.2962 (0.2687,0.3265) |
|  | 30-34 | 686 | 0.58 |  | C | 0.3340 (0.3097,0.3602) |  |  | B | 0.3344 (0.3091,0.3618) |
|  | 35+ | 663 | 0.60 |  | D | 0.4396 (0.4020,0.4807) |  |  | C | 0.4343 (0.4008,0.4707) |
| Oxychlordane | <25 | 137 | 31.39 | <.0001 | A | 0.0065 (0.0058,0.0072) | <.0001 | <.0001 | A | 0.0067 (0.0059,0.0075) |
|  | 25-29 | 448 | 8.48 |  | B | 0.0105 (0.0098,0.0111) |  |  | B | 0.0106 (0.0099,0.0113) |
|  | 30-34 | 685 | 5.40 |  | C | 0.0126 (0.0120,0.0132) |  |  | C | 0.0126 (0.0120,0.0133) |
|  | 35+ | 663 | 4.98 |  | D | 0.0153 (0.0144,0.0162) |  |  | D | 0.0150 (0.0143,0.0158) |
| PBDE47 | <25 | 138 | 32.61 | 0.0099 | A | 0.0436 (0.0341,0.0557) | <.0001 | 0.0017 | A | 0.0457 (0.0359,0.0583) |
|  | 25-29 | 447 | 29.53 |  | A | 0.0498 (0.0432,0.0573) |  |  | A | 0.0507 (0.0444,0.0580) |
|  | 30-34 | 683 | 35.72 |  | A | 0.0410 (0.0366,0.0460) |  |  | A | 0.0410 (0.0367,0.0458) |
|  | 35+ | 660 | 36.36 |  | A | 0.0410 (0.0364,0.0461) |  |  | A | 0.0400 (0.0357,0.0448) |
| Arochlor1260 | <25 | 138 | 21.74 | <.0001 | A | 0.1613 (0.1400,0.1858) | <.0001 | <.0001 | A | 0.1648 (0.1422,0.1910) |
|  | 25-29 | 448 | 3.13 |  | B | 0.2800 (0.2578,0.3042) |  |  | B | 0.2819 (0.2603,0.3053) |
|  | 30-34 | 686 | 0.73 |  | C | 0.3609 (0.3394,0.3838) |  |  | C | 0.3613 (0.3388,0.3853) |
|  | 35+ | 663 | 0.45 |  | D | 0.5333 (0.4980,0.5710) |  |  | D | 0.5271 (0.4936,0.5628) |
| PCB138 | <25 | 138 | 35.51 | <.0001 | A | 0.0122 (0.0105,0.0142) | <.0001 | <.0001 | A | 0.0125 (0.0107,0.0145) |
|  | 25-29 | 448 | 10.49 |  | B | 0.0200 (0.0184,0.0217) |  |  | B | 0.0202 (0.0187,0.0219) |
|  | 30-34 | 686 | 3.79 |  | C | 0.0256 (0.0241,0.0272) |  |  | C | 0.0256 (0.0240,0.0273) |
|  | 35+ | 663 | 2.11 |  | D | 0.0361 (0.0337,0.0386) |  |  | D | 0.0356 (0.0333,0.0380) |
| PCB153 | <25 | 138 | 13.04 | <.0001 | A | 0.0190 (0.0165,0.0218) | <.0001 | <.0001 | A | 0.0194 (0.0167,0.0224) |
|  | 25-29 | 448 | 1.12 |  | B | 0.0337 (0.0310,0.0366) |  |  | B | 0.0339 (0.0313,0.0367) |
|  | 30-34 | 686 | 0.15 |  | C | 0.0437 (0.0411,0.0465) |  |  | C | 0.0438 (0.0410,0.0467) |
|  | 35+ | 663 | 0.15 |  | D | 0.0662 (0.0617,0.0709) |  |  | D | 0.0655 (0.0613,0.0700) |
| PCB180 | <25 | 138 | 47.83 | <.0001 | A | 0.0102 (0.0085,0.0121) | <.0001 | <.0001 | A | 0.0101 (0.0085,0.0120) |
|  | 25-29 | 448 | 12.05 |  | B | 0.0212 (0.0193,0.0233) |  |  | B | 0.0214 (0.0196,0.0234) |
|  | 30-34 | 686 | 2.92 |  | C | 0.0292 (0.0272,0.0312) |  |  | C | 0.0292 (0.0272,0.0313) |
|  | 35+ | 663 | 0.45 |  | D | 0.0478 (0.0445,0.0514) |  |  | D | 0.0474 (0.0441,0.0510) |
| PFHxS | <25 | 138 | 2.90 | <.0001 | A | 1.2908 (1.0447,1.5949) |  |  |  |  |
|  | 25-29 | 445 | 4.27 |  | A | 1.0987 (0.9914,1.2175) |  |  |  |  |
|  | 30-34 | 694 | 3.46 |  | AB | 1.0581 (0.9808,1.1415) |  |  |  |  |
|  | 35+ | 663 | 4.98 |  | B | 0.9139 (0.8454,0.9879) |  |  |  |  |
| PFOA | <25 | 138 | 0.72 | <.0001 | AB | 1.7123 (1.5123,1.9387) |  |  |  |  |
|  | 25-29 | 445 | 0.22 |  | A | 1.8105 (1.6851,1.9451) |  |  |  |  |
|  | 30-34 | 694 | 0.14 |  | AB | 1.6744 (1.5772,1.7777) |  |  |  |  |
|  | 35+ | 663 | 0.00 |  | B | 1.5062 (1.4192,1.5985) |  |  |  |  |
| PFOS | <25 | 138 | 1.45 | 0.0413** | A | 4.500 (3.9531, 5.0469) |  |  |  |  |
|  | 25-29 | 445 | 0.22 |  | A | 4.900 (4.4895, 5.3105) |  |  |  |  |
|  | 30-34 | 694 | 0.00 |  | A | 4.800 (4.4610, 5.1390) |  |  |  |  |
|  | 35+ | 663 | 0.00 |  | A | 4.500 (4.2170, 4.7830) |  |  |  |  |

^*^ 95% CI: the 95% confidence intervals for the geometric mean were corrected for multiple comparisons using Bonferroni correction

^**^ The analysis is based on the non-parametric Wilcoxon test and Kaplan-Meier estimates of the median. Confidence intervals were calculated using the Greenwood variance estimate.

# Table S3: Results for MIREC persistent organic pollutants in maternal blood by smoking status (µg/L)

| **Contaminant** | **Group** | **N** | **%<LOD** | **Unadjusted** | | | **lipid as a covariate** | | | |
| --- | --- | --- | --- | --- | --- | --- | --- | --- | --- | --- |
|  |  |  |  | **P-Value** | **GM* (95% CI)** | | **P-Value for lipids** | **P-Value** | **GM* (95% CI)** | |
| Beta-HCH | Current | 230 | 41.30 | 0.0002 | A | 0.0114 (0.0097, 0.0135) | <.0001 | <.0001 | A | 0.0099 (0.0081, 0.0122) |
|  | Former | 515 | 27.38 |  | B | 0.0153 (0.0137, 0.0172) |  |  | B | 0.0145 (0.0127, 0.0165) |
|  | Never | 1151 | 32.06 |  | AB | 0.0145 (0.0131, 0.0161) |  |  | B | 0.0151 (0.0138, 0.0165) |
| DDE | Current | 233 | 3.00 | <.0001 | A | 0.2814 (0.2548, 0.3107) | <.0001 | <.0001 | A | 0.2786 (0.2455, 0.3161) |
|  | Former | 529 | 0.57 |  | AB | 0.3321 (0.3098, 0.3560) |  |  | AB | 0.3308 (0.3042, 0.3597) |
|  | Never | 1171 | 0.85 |  | B | 0.3622 (0.3401, 0.3857) |  |  | B | 0.3633 (0.3435, 0.3844) |
| Oxychlordane | Current | 233 | 10.73 | 0.0002 | A | 0.0111 (0.0102, 0.0122) | <.0001 | 0.0001 | A | 0.0111 (0.0102, 0.0121) |
|  | Former | 528 | 6.63 |  | B | 0.0133 (0.0125, 0.0141) |  |  | B | 0.0132 (0.0125, 0.0140) |
|  | Never | 1170 | 7.78 |  | AB | 0.0121 (0.0116, 0.0126) |  |  | AB | 0.0121 (0.0117, 0.0126) |
| PBDE153++ | Current | 232 | 44.40 | 0.0024 | A | 0.0231 (0.0179, 0.0298) | 0.0063 | 0.0028 | A | 0.0219 (0.0169, 0.0284) |
|  | Former | 529 | 56.14 |  | AB | 0.0153 (0.0123, 0.0189) |  |  | AB | 0.0156 (0.0129, 0.0188) |
|  | Never | 1165 | 57.60 |  | B | 0.0144 (0.0125, 0.0167) |  |  | B | 0.0145 (0.0127, 0.0166) |
| PBDE47 | Current | 232 | 32.76 | 0.0173 | A | 0.0493 (0.0404, 0.0602) | <.0001 | 0.0164 | A | 0.0509 (0.0429, 0.0602) |
|  | Former | 529 | 35.92 |  | A | 0.0405 (0.0361, 0.0454) |  |  | A | 0.0399 (0.0355, 0.0447) |
|  | Never | 1165 | 33.82 |  | A | 0.0432 (0.0400, 0.0467) |  |  | A | 0.0432 (0.0400, 0.0467) |
| Arochlor1260 | Current | 233 | 6.44 | <.0001 | A | 0.2844 (0.2552, 0.3169) | <.0001 | <.0001 | A | 0.2820 (0.2523, 0.3153) |
|  | Former | 529 | 1.51 |  | B | 0.4059 (0.3760, 0.4382) |  |  | B | 0.4045 (0.3758, 0.4353) |
|  | Never | 1171 | 2.48 |  | B | 0.3698 (0.3518, 0.3888) |  |  | B | 0.3709 (0.3530, 0.3897) |
| PCB118 | Current | 233 | 38.20 | <.0001 | A | 0.0118 (0.0106, 0.0132) | <.0001 | <.0001 | A | 0.0117 (0.0106, 0.0130) |
|  | Former | 529 | 24.76 |  | B | 0.0152 (0.0141, 0.0163) |  |  | B | 0.0151 (0.0141, 0.0161) |
|  | Never | 1171 | 25.19 |  | B | 0.0148 (0.0141, 0.0155) |  |  | B | 0.0148 (0.0142, 0.0155) |
| PCB138 | Current | 233 | 12.88 | <.0001 | A | 0.0209 (0.0188, 0.0232) | <.0001 | <.0001 | A | 0.0207 (0.0185, 0.0231) |
|  | Former | 529 | 5.86 |  | B | 0.0281 (0.0260, 0.0303) |  |  | B | 0.0280 (0.0261, 0.0301) |
|  | Never | 1171 | 6.40 |  | B | 0.0259 (0.0247, 0.0272) |  |  | B | 0.0260 (0.0248, 0.0273) |
| PCB153 | Current | 233 | 4.29 | <.0001 | A | 0.0339 (0.0304, 0.0378) | <.0001 | <.0001 | A | 0.0337 (0.0301, 0.0377) |
|  | Former | 529 | 0.76 |  | B | 0.0496 (0.0459, 0.0537) |  |  | B | 0.0495 (0.0459, 0.0533) |
|  | Never | 1171 | 0.94 |  | B | 0.0451 (0.0428, 0.0474) |  |  | B | 0.0452 (0.0430, 0.0475) |
| PCB180 | Current | 233 | 15.02 | <.0001 | A | 0.0213 (0.0189, 0.0240) | <.0001 | <.0001 | A | 0.0210 (0.0185, 0.0239) |
|  | Former | 529 | 4.91 |  | B | 0.0338 (0.0310, 0.0368) |  |  | B | 0.0337 (0.0310, 0.0366) |
|  | Never | 1171 | 7.00 |  | B | 0.0303 (0.0286, 0.0321) |  |  | B | 0.0304 (0.0287, 0.0321) |
| Transnonachlor | Current | 233 | 21.46 | 0.0004 | A | 0.0155 (0.0141, 0.0171) | <.0001 | 0.0002 | A | 0.0154 (0.0140, 0.0169) |
|  | Former | 529 | 15.12 |  | B | 0.0188 (0.0177, 0.0200) |  |  | B | 0.0187 (0.0176, 0.0199) |
|  | Never | 1170 | 15.13 |  | B | 0.0178 (0.0171, 0.0186) |  |  | B | 0.0179 (0.0172, 0.0187) |
| PFHxS | Current | 235 | 2.13 | 0.0042 | A | 1.1873 (1.0456, 1.3482) |  |  |  |  |
|  | Former | 528 | 3.98 |  | A | 1.0522 (0.9707, 1.1405) |  |  |  |  |
|  | Never | 1175 | 4.60 |  | A | 0.9894 (0.9359, 1.0460) |  |  |  |  |
| PFOA | Current | 235 | 0.00 | 0.0269 | A | 1.7897 (1.6393, 1.9540) |  |  |  |  |
|  | Former | 528 | 0.19 |  | A | 1.6735 (1.5740, 1.7793) |  |  |  |  |
|  | Never | 1175 | 0.17 |  | A | 1.6063 (1.5415, 1.6740) |  |  |  |  |
| PFOS | Current | 235 | 0.00 | 0.6769 |  | 4.4235 (4.0686, 4.8093) |  |  |  |  |
|  | Former | 528 | 0.19 |  |  | 4.5619 (4.2889, 4.8522) |  |  |  |  |
|  | Never | 1175 | 0.17 |  |  | 4.5884 (4.4054, 4.7790) |  |  |  |  |

^*^ 95% CI: the 95% confidence intervals for the geometric mean were corrected for multiple comparisons using Bonferroni correction

++ Caution should be taken since two groups have more than 50% observations undetected.

# Table S4: Results for MIREC persistent organic pollutants in maternal blood by household income (µg/L)

| **Contaminant** | **Group** | **N** | **%<LOD** | **Unadjusted** | | | **lipid as a covariate** | | | |
| --- | --- | --- | --- | --- | --- | --- | --- | --- | --- | --- |
|  |  |  |  | **P-Value** | **GM* (95% CI)** | | **P-Value for lipids** | **P-Value** | **GM* (95% CI)** | |
| Beta-HCH | <=$50,000 | 333 | 40.24 | 0.0041 | A | 0.0116 (0.0092, 0.0146) | <.0001 | 0.0013 | AB | 0.0132 (0.0112, 0.0156) |
|  | $50,001-$100,000 | 749 | 33.51 |  | AB | 0.0135 (0.0121, 0.0150) |  |  | AB | 0.0130 (0.0116, 0.0145) |
|  | More than $100,000 | 724 | 25.41 |  | B | 0.0164 (0.0147, 0.0182) |  |  | B | 0.0162 (0.0145, 0.0181) |
| DDE | <=$50,000 | 339 | 3.24 | 0.0214 | A | 0.3356 (0.2920, 0.3858) | <.0001 | 0.0112 | A | 0.3388 (0.3051, 0.3762) |
|  | $50,001-$100,000 | 764 | 0.65 |  | A | 0.3254 (0.3046, 0.3476) |  |  | A | 0.3236 (0.3018, 0.3470) |
|  | More than $100,000 | 740 | 0.41 |  | A | 0.3651 (0.3425, 0.3891) |  |  | A | 0.3663 (0.3413, 0.3932) |
| Oxychlordane | <=$50,000 | 338 | 15.38 | <.0001 | A | 0.0104 (0.0095, 0.0113) | <.0001 | <.0001 | A | 0.0106 (0.0099, 0.0114) |
|  | $50,001-$100,000 | 763 | 7.34 |  | B | 0.0118 (0.0113, 0.0124) |  |  | A | 0.0117 (0.0112, 0.0123) |
|  | More than $100,000 | 740 | 4.05 |  | C | 0.0140 (0.0134, 0.0147) |  |  | B | 0.0141 (0.0135, 0.0148) |
| PBDE47 | <=$50,000 | 337 | 35.31 | 0.0190 | A | 0.0420 (0.0361, 0.0490) | <.0001 | 0.0329 | A | 0.0426 (0.0369, 0.0492) |
|  | $50,001-$100,000 | 762 | 31.76 |  | A | 0.0464 (0.0421, 0.0513) |  |  | A | 0.0463 (0.0421, 0.0509) |
|  | More than $100,000 | 737 | 36.64 |  | A | 0.0401 (0.0363, 0.0443) |  |  | A | 0.0399 (0.0362, 0.0441) |
| Arochlor1260 | <=$50,000 | 339 | 7.96 | <.0001 | A | 0.2880 (0.2584, 0.3211) | <.0001 | <.0001 | A | 0.2912 (0.2659, 0.3189) |
|  | $50,001-$100,000 | 764 | 2.62 |  | B | 0.3516 (0.3303, 0.3743) |  |  | B | 0.3496 (0.3292, 0.3714) |
|  | More than $100,000 | 740 | 0.27 |  | C | 0.4337 (0.4103, 0.4584) |  |  | C | 0.4354 (0.4095, 0.4628) |
| PCB118 | <=$50,000 | 339 | 37.76 | <.0001 | A | 0.0121 (0.0109, 0.0134) | <.0001 | <.0001 | A | 0.0124 (0.0114, 0.0136) |
|  | $50,001-$100,000 | 764 | 29.19 |  | A | 0.0139 (0.0130, 0.0147) |  |  | A | 0.0139 (0.0131, 0.0147) |
|  | More than $100,000 | 740 | 17.84 |  | B | 0.0166 (0.0157, 0.0174) |  |  | B | 0.0165 (0.0156, 0.0174) |
| PCB138 | <=$50,000 | 339 | 15.93 | <.0001 | A | 0.0207 (0.0186, 0.0230) | <.0001 | <.0001 | A | 0.0211 (0.0193, 0.0230) |
|  | $50,001-$100,000 | 764 | 6.81 |  | B | 0.0248 (0.0233, 0.0264) |  |  | B | 0.0247 (0.0232, 0.0261) |
|  | More than $100,000 | 740 | 2.84 |  | C | 0.0300 (0.0284, 0.0317) |  |  | C | 0.0301 (0.0283, 0.0319) |
| PCB153 | <=$50,000 | 339 | 5.31 | <.0001 | A | 0.0345 (0.0309, 0.0385) | <.0001 | <.0001 | A | 0.0348 (0.0317, 0.0382) |
|  | $50,001-$100,000 | 764 | 0.65 |  | B | 0.0428 (0.0402, 0.0456) |  |  | B | 0.0426 (0.0400, 0.0453) |
|  | More than $100,000 | 740 | 0.00 |  | C | 0.0532 (0.0503, 0.0563) |  |  | C | 0.0534 (0.0502, 0.0568) |
| PCB180 | <=$50,000 | 339 | 19.17 | <.0001 | A | 0.0213 (0.0187, 0.0243) | <.0001 | <.0001 | A | 0.0220 (0.0198, 0.0244) |
|  | $50,001-$100,000 | 764 | 6.94 |  | B | 0.0286 (0.0267, 0.0307) |  |  | B | 0.0285 (0.0266, 0.0305) |
|  | More than $100,000 | 740 | 1.62 |  | C | 0.0366 (0.0344, 0.0390) |  |  | C | 0.0367 (0.0343, 0.0393) |
| Transnonachlor | <=$50,000 | 338 | 29.59 | <.0001 | A | 0.0143 (0.0129, 0.0159) | <.0001 | <.0001 | A | 0.0149 (0.0138, 0.0161) |
|  | $50,001-$100,000 | 764 | 16.36 |  | B | 0.0170 (0.0162, 0.0179) |  |  | A | 0.0169 (0.0160, 0.0177) |
|  | More than $100,000 | 740 | 7.57 |  | C | 0.0209 (0.0199, 0.0219) |  |  | B | 0.0209 (0.0199, 0.0220) |
| PFHxS | <=$50,000 | 339 | 6.19 | 0.0119** | A | 0.930 (0.8027, 1.0573) |  |  |  |  |
|  | $50,001-$100,000 | 763 | 3.80 |  | A | 1.000 (0.9182, 1.0818) |  |  |  |  |
|  | More than $100,000 | 745 | 3.09 |  | A | 1.000 (0.9065, 1.0935) |  |  |  |  |
| PFOA | <=$50,000 | 339 | 0.29 | 0.0005 | A | 1.5218 (1.4099, 1.6425) |  |  |  |  |
|  | $50,001-$100,000 | 763 | 0.13 |  | AB | 1.6213 (1.5393, 1.7076) |  |  |  |  |
|  | More than $100,000 | 745 | 0.00 |  | B | 1.7521 (1.6679, 1.8405) |  |  |  |  |
| PFOS | <=$50,000 | 339 | 0.59 | <.0001 | A | 4.0647 (3.7376, 4.4203) |  |  |  |  |
|  | $50,001-$100,000 | 763 | 0.13 |  | AB | 4.5479 (4.3286, 4.7784) |  |  |  |  |
|  | More than $100,000 | 745 | 0.00 |  | B | 4.8889 (4.6574, 5.1319) |  |  |  |  |

^*^ 95% CI: the 95% confidence intervals for the geometric mean were corrected for multiple comparisons using Bonferroni correction

^**^ The analysis is based on the non-parametric Wilcoxon test and Kaplan-Meier estimates of the median. Confidence intervals were calculated using the Greenwood variance estimate.

# Table S5: Results for MIREC persistent organic pollutants in maternal blood by pre-BMI (µg/L)

| **Contaminant** | **Group** | **N** | **%<LOD** | **Unadjusted** | | | **lipid as a covariate** | | | |
| --- | --- | --- | --- | --- | --- | --- | --- | --- | --- | --- |
|  |  |  |  | **P-Value** | **GM* (95% CI)** | | **P-Value for lipids** | **P-Value** | **GM* (95% CI)** | |
| Beta-HCH | Underweight to Normal (BMI < 25) | 1111 | 35.46 | <.0001** | A | 0.013 (0.0122, 0.0138) | 0.0309*** | |  |  |
|  | Overweight (25 <= BMI < 30) | 394 | 24.62 |  | AB | 0.015 (0.0137, 0.0163) |  |  |  |  |
|  | Obese (BMI >=30) | 258 | 22.09 |  | B | 0.017 (0.0150, 0.0190) |  |  |  |  |
| DDE | Underweight to Normal (BMI < 25) | 1134 | 0.79 | 0.0029 | A | 0.3609 (0.3405, 0.3825) | 0.0053*** | |  |  |
|  | Overweight (25 <= BMI < 30) | 399 | 1.00 |  | AB | 0.3359 (0.3059, 0.3688) |  |  |  |  |
|  | Obese (BMI >=30) | 263 | 0.76 |  | B | 0.3008 (0.2683, 0.3372) |  |  |  |  |
| Oxychlordane | Underweight to Normal (BMI < 25) | 1133 | 6.80 | 0.0111 | A | 0.0128 (0.0123, 0.0133) | <.0001 | <.0001 | A | 0.0132 (0.0127, 0.0137) |
|  | Overweight (25 <= BMI < 30) | 399 | 7.52 |  | A | 0.0123 (0.0115, 0.0132) |  |  | AB | 0.0120 (0.0112, 0.0128) |
|  | Obese (BMI >=30) | 263 | 9.51 |  | A | 0.0114 (0.0105, 0.0124) |  |  | B | 0.0105 (0.0097, 0.0114) |
| PBDE47 | Underweight to Normal (BMI < 25) | 1130 | 40.00 | <.0001 | A | 0.0372 (0.0342, 0.0405) | <.0001 | <.0001 | A | 0.0384 (0.0354, 0.0416) |
|  | Overweight (25 <= BMI < 30) | 397 | 29.22 |  | B | 0.0467 (0.0413, 0.0528) |  |  | A | 0.0452 (0.0398, 0.0514) |
|  | Obese (BMI >=30) | 263 | 19.39 |  | C | 0.0666 (0.0573, 0.0774) |  |  | B | 0.0627 (0.0537, 0.0731) |
| Arochlor1260 | Underweight to Normal (BMI < 25) | 1134 | 1.59 | <.0001 | A | 0.4053 (0.3853, 0.4264) | <.0001 | <.0001 | A | 0.4155 (0.3955, 0.4366) |
|  | Overweight (25 <= BMI < 30) | 399 | 2.26 |  | A | 0.3597 (0.3296, 0.3927) |  |  | B | 0.3513 (0.3234, 0.3817) |
|  | Obese (BMI >=30) | 263 | 4.18 |  | B | 0.2816 (0.2564, 0.3093) |  |  | C | 0.2615 (0.2358, 0.2900) |
| PCB118 | Underweight to Normal (BMI < 25) | 1134 | 26.37 | 0.3867 |  | 0.0148 (0.0140, 0.0155) | <.0001 | 0.0027 | A | 0.0152 (0.0145, 0.0159) |
|  | Overweight (25 <= BMI < 30) | 399 | 23.56 |  |  | 0.0151 (0.0139, 0.0163) |  |  | AB | 0.0146 (0.0135, 0.0158) |
|  | Obese (BMI >=30) | 263 | 25.86 |  |  | 0.0141 (0.0129, 0.0155) |  |  | B | 0.0130 (0.0118, 0.0143) |
| PCB138 | Underweight to Normal (BMI < 25) | 1134 | 5.03 | <.0001 | A | 0.0278 (0.0264, 0.0292) | <.0001 | <.0001 | A | 0.0285 (0.0272, 0.0299) |
|  | Overweight (25 <= BMI < 30) | 399 | 7.77 |  | A | 0.0257 (0.0235, 0.0280) |  |  | B | 0.0251 (0.0231, 0.0272) |
|  | Obese (BMI >=30) | 263 | 9.89 |  | B | 0.0211 (0.0192, 0.0232) |  |  | C | 0.0195 (0.0176, 0.0216) |
| PCB153 | Underweight to Normal (BMI < 25) | 1134 | 0.71 | <.0001 | A | 0.0500 (0.0474, 0.0526) | <.0001 | <.0001 | A | 0.0512 (0.0487, 0.0538) |
|  | Overweight (25 <= BMI < 30) | 399 | 0.50 |  | B | 0.0434 (0.0398, 0.0474) |  |  | B | 0.0424 (0.0390, 0.0461) |
|  | Obese (BMI >=30) | 263 | 3.42 |  | C | 0.0328 (0.0298, 0.0361) |  |  | C | 0.0305 (0.0274, 0.0339) |
| PCB180 | Underweight to Normal (BMI < 25) | 1134 | 3.62 | <.0001 | A | 0.0348 (0.0329, 0.0369) | <.0001 | <.0001 | A | 0.0357 (0.0338, 0.0377) |
|  | Overweight (25 <= BMI < 30) | 399 | 7.77 |  | B | 0.0280 (0.0254, 0.0309) |  |  | B | 0.0274 (0.0250, 0.0301) |
|  | Obese (BMI >=30) | 263 | 17.11 |  | C | 0.0195 (0.0175, 0.0217) |  |  | C | 0.0179 (0.0160, 0.0202) |
| Transnonachlor | Underweight to Normal (BMI < 25) | 1134 | 13.58 | 0.0006 | A | 0.0188 (0.0180, 0.0196) | <.0001 | <.0001 | A | 0.0194 (0.0186, 0.0202) |
|  | Overweight (25 <= BMI < 30) | 399 | 15.29 |  | AB | 0.0177 (0.0164, 0.0190) |  |  | B | 0.0172 (0.0160, 0.0184) |
|  | Obese (BMI >=30) | 263 | 21.29 |  | B | 0.0160 (0.0146, 0.0176) |  |  | B | 0.0148 (0.0135, 0.0162) |
| PFHxS | Underweight to Normal (BMI < 25) | 1142 | 4.82 | 0.0365 | A | 0.9875 (0.9329, 1.0452) |  |  |  |  |
|  | Overweight (25 <= BMI < 30) | 394 | 4.31 |  | A | 1.0929 (0.9975, 1.1973) |  |  |  |  |
|  | Obese (BMI >=30) | 262 | 2.67 |  | A | 1.0907 (0.9698, 1.2266) |  |  |  |  |
| PFOA | Underweight to Normal (BMI < 25) | 1142 | 0.09 | 0.8120 |  | 1.6520 (1.5847, 1.7222) |  |  |  |  |
|  | Overweight (25 <= BMI < 30) | 394 | 0.25 |  |  | 1.6344 (1.5194, 1.7580) |  |  |  |  |
|  | Obese (BMI >=30) | 262 | 0.38 |  |  | 1.6113 (1.4820, 1.7519) |  |  |  |  |
| PFOS | Underweight to Normal (BMI < 25) | 1142 | 0.18 | 0.5110 |  | 4.6083 (4.4177, 4.8071) |  |  |  |  |
|  | Overweight (25 <= BMI < 30) | 394 | 0.00 |  |  | 4.5827 (4.2784, 4.9088) |  |  |  |  |
|  | Obese (BMI >=30) | 262 | 0.38 |  |  | 4.4005 (4.0577, 4.7722) |  |  |  |  |

^*^ 95% CI: the 95% confidence intervals for the geometric mean were corrected for multiple comparisons using Bonferroni correction

^**^ The analysis is based on the non-parametric Wilcoxon test and Kaplan-Meier estimates of the median. Confidence intervals were calculated using the Greenwood variance estimate.

***Interaction between lipids and pre-pregnancy BMI was significant – see Table 9.

# Table S6: Results for MIREC persistent organic pollutants in maternal blood by place of birth (µg/L)

| **Contaminant** | **Group** | **N** | **%<LOD** | **Unadjusted** | | | **lipid as a covariate** | | | |
| --- | --- | --- | --- | --- | --- | --- | --- | --- | --- | --- |
|  |  |  |  | **P-Value** | **GM* (95% CI)** | | **P-Value for lipids** | **P-Value** | **GM* (95% CI)** | |
| Beta-HCH | Foreign born | 358 | 12.85 | <.0001 | A | 0.0474 (0.0399, 0.0563) | <.0001 | <.0001 | A | 0.0504 (0.0455, 0.0558) |
|  | Canadian born | 1540 | 36.30 |  | B | 0.0124 (0.0119, 0.0128) |  |  | B | 0.0111 (0.0105, 0.0117) |
| DDE | Foreign born | 360 | 0.28 | <.0001 | A | 0.8388 (0.7475, 0.9413) | <.0001 | <.0001 | A | 0.8401 (0.7828, 0.9016) |
|  | Canadian born | 1575 | 1.21 |  | B | 0.2801 (0.2725, 0.2878) |  |  | B | 0.2797 (0.2704, 0.2893) |
| Oxychlordane | Foreign born | 360 | 10.28 | 0.0003 | A | 0.0134 (0.0125, 0.0145) | <.0001 | 0.0001 | A | 0.0136 (0.0128, 0.0144) |
|  | Canadian born | 1573 | 7.25 |  | B | 0.0120 (0.0117, 0.0124) |  |  | B | 0.0120 (0.0117, 0.0123) |
| PBDE47 | Foreign born | 360 | 45.00 | <.0001 | A | 0.0331 (0.0288, 0.0380) | <.0001 | <.0001 | A | 0.0347 (0.0308, 0.0389) |
|  | Canadian born | 1568 | 31.82 |  | B | 0.0454 (0.0430, 0.0480) |  |  | B | 0.0452 (0.0428, 0.0477) |
| Arochlor1260 | Foreign born | 360 | 0.83 | <.0001 | A | 0.6336 (0.5796, 0.6926) | <.0001 | <.0001 | A | 0.6347 (0.5927, 0.6797) |
|  | Canadian born | 1575 | 3.11 |  | B | 0.3250 (0.3151, 0.3351) |  |  | B | 0.3245 (0.3141, 0.3353) |
| PCB118 | Foreign born | 360 | 14.17 | <.0001 | A | 0.0197 (0.0183, 0.0212) | <.0001 | <.0001 | A | 0.0198 (0.0186, 0.0211) |
|  | Canadian born | 1575 | 29.46 |  | B | 0.0136 (0.0132, 0.0140) |  |  | B | 0.0135 (0.0131, 0.0139) |
| PCB138 | Foreign born | 360 | 3.06 | <.0001 | A | 0.0423 (0.0388, 0.0460) | <.0001 | <.0001 | A | 0.0424 (0.0397, 0.0454) |
|  | Canadian born | 1575 | 7.94 |  | B | 0.0231 (0.0225, 0.0239) |  |  | B | 0.0231 (0.0223, 0.0238) |
| PCB153 | Foreign born | 360 | 0.00 | <.0001 | A | 0.0793 (0.0724, 0.0869) | <.0001 | <.0001 | A | 0.0794 (0.0741, 0.0851) |
|  | Canadian born | 1575 | 1.59 |  | B | 0.0392 (0.0380, 0.0405) |  |  | B | 0.0392 (0.0379, 0.0405) |
| PCB170++ | Foreign born | 360 | 23.33 | <.0001 | A | 0.0202 (0.0181, 0.0226) | <.0001 | <.0001 | A | 0.0212 (0.0195, 0.0231) |
|  | Canadian born | 1575 | 52.19 |  | B | 0.0096 (0.0092, 0.0100) |  |  | B | 0.0090 (0.0086, 0.0094) |
| PCB180 | Foreign born | 360 | 3.33 | <.0001 | A | 0.0555 (0.0500, 0.0617) | <.0001 | <.0001 | A | 0.0559 (0.0517, 0.0604) |
|  | Canadian born | 1575 | 8.32 |  | B | 0.0261 (0.0252, 0.0270) |  |  | B | 0.0260 (0.0250, 0.0269) |
| Transnonachlor | Foreign born | 360 | 18.33 | <.0001 | A | 0.0197 (0.0182, 0.0213) | <.0001 | <.0001 | A | 0.0201 (0.0189, 0.0214) |
|  | Canadian born | 1574 | 15.31 |  | B | 0.0174 (0.0169, 0.0179) |  |  | B | 0.0173 (0.0168, 0.0178) |
| PFHxS | Foreign born | 361 | 9.14 | <.0001 | A | 0.7459 (0.6874, 0.8094) |  |  |  |  |
|  | Canadian born | 1579 | 2.98 |  | B | 1.1077 (1.0661, 1.1510) |  |  |  |  |
| PFOA | Foreign born | 361 | 0.28 | 0.2384 |  | 1.5934 (1.4986, 1.6943) |  |  |  |  |
|  | Canadian born | 1579 | 0.13 |  |  | 1.6591 (1.6119, 1.7078) |  |  |  |  |
| PFOS | Foreign born | 361 | 0.00 | 0.0004 | A | 4.1410 (3.8825, 4.4167) |  |  |  |  |
|  | Canadian born | 1579 | 0.19 |  | B | 4.6666 (4.5378, 4.7989) |  |  |  |  |

^*^ 95% CI: the 95% confidence intervals for the geometric mean were corrected for multiple comparisons using Bonferroni correction

++ Caution should be taken since one group has more than 50% observations undetected.

# Table S7: Results for MIREC persistent organic pollutants in maternal blood by fasting (µg/L)

| **Contaminant** | **Group** | **N** | **%<LOD** | **Unadjusted** | | | **lipid as a covariate** | | | |
| --- | --- | --- | --- | --- | --- | --- | --- | --- | --- | --- |
|  |  |  |  | **P-Value** | **GM* (95% CI)** | | **P-Value for lipids** | **P-Value** | **GM* (95% CI)** | |
| Beta-HCH | No | 1856 | 31.79 | 0.1128 |  | 0.0143 (0.0135, 0.0152) | <.0001 | 0.1711 |  | 0.0143 (0.0135, 0.0151) |
|  | Yes | 39 | 35.90 |  |  | 0.0124 (0.0099, 0.0155) |  |  |  | 0.0107 (0.0072, 0.0161) |
| DDE | No | 1893 | 1.06 | 0.0194 | A | 0.3452 (0.3326, 0.3582) | <.0001 | 0.0266 | A | 0.3450 (0.3327, 0.3578) |
|  | Yes | 39 | 0.00 |  | B | 0.2535 (0.2112, 0.3042) |  |  | B | 0.2582 (0.2003, 0.3327) |
| Oxychlordane | No | 1891 | 7.62 | 0.0033 | A | 0.0123 (0.0120, 0.0127) | <.0001 | 0.0064 | A | 0.0123 (0.0120, 0.0127) |
|  | Yes | 39 | 17.95 |  | B | 0.0093 (0.0076, 0.0113) |  |  | B | 0.0096 (0.0081, 0.0115) |
| PBDE153++ | No | 1886 | 55.67 | 0.9089 |  | 0.0155 (0.0142, 0.0170) | 0.0062 | 0.8620 |  | 0.0156 (0.0142, 0.0170) |
|  | Yes | 39 | 53.85 |  |  | 0.0176 (0.0102, 0.0304) |  |  |  | 0.0163 (0.0095, 0.0279) |
| PBDE47 | No | 1886 | 34.41 | 0.2369 |  | 0.0429 (0.0408, 0.0452) | <.0001 | 0.1747 |  | 0.0429 (0.0407, 0.0451) |
|  | Yes | 39 | 28.21 |  |  | 0.0509 (0.0343, 0.0757) |  |  |  | 0.0542 (0.0388, 0.0758) |
| Arochlor1260 | No | 1893 | 2.64 | 0.0356 | A | 0.3692 (0.3573, 0.3814) | <.0001 | 0.0502 |  | 0.3690 (0.3573, 0.3810) |
|  | Yes | 39 | 5.13 |  | A | 0.2878 (0.2249, 0.3682) |  |  |  | 0.2941 (0.2349, 0.3682) |
| PCB118 | No | 1893 | 26.41 | 0.0596 |  | 0.0146 (0.0141, 0.0150) | <.0001 | 0.0911 |  | 0.0145 (0.0141, 0.0150) |
|  | Yes | 39 | 38.46 |  |  | 0.0117 (0.0093, 0.0148) |  |  |  | 0.0121 (0.0098, 0.0149) |
| PCB138 | No | 1893 | 6.97 | 0.0232 | A | 0.0259 (0.0251, 0.0268) | <.0001 | 0.0348 | A | 0.0259 (0.0251, 0.0268) |
|  | Yes | 39 | 10.26 |  | B | 0.0199 (0.0158, 0.0251) |  |  | B | 0.0204 (0.0164, 0.0254) |
| PCB153 | No | 1893 | 1.32 | 0.0294** | A | 0.043 (0.0413, 0.0447) | <.0001 | 0.0830 |  | 0.0449 (0.0434, 0.0464) |
|  | Yes | 39 | 0.00 |  | B | 0.030 (0.0219, 0.0381) |  |  |  | 0.0366 (0.0291, 0.0460) |
| PCB180 | No | 1893 | 7.29 | 0.0305** | A | 0.029 (0.0279, 0.0301) | <.0001 | 0.0670 |  | 0.0300 (0.0289, 0.0311) |
|  | Yes | 39 | 12.82 |  | B | 0.021 (0.0159, 0.0261) |  |  |  | 0.0235 (0.0182, 0.0304) |
| Transnonachlor | No | 1892 | 15.80 | 0.0157 | A | 0.0179 (0.0174, 0.0184) | <.0001 | 0.0271 | A | 0.0179 (0.0174, 0.0184) |
|  | Yes | 39 | 20.51 |  | B | 0.0140 (0.0115, 0.0171) |  |  | B | 0.0144 (0.0120, 0.0174) |
| PFHxS | No | 1898 | 4.06 | 0.4918 |  | 1.0307 (0.9946, 1.0682) |  |  |  |  |
|  | Yes | 39 | 7.69 |  |  | 0.9401 (0.7176, 1.2317) |  |  |  |  |
| PFOA | No | 1898 | 0.16 | 0.7584 |  | 1.6456 (1.6027, 1.6896) |  |  |  |  |
|  | Yes | 39 | 0.00 |  |  | 1.6944 (1.3890, 2.0671) |  |  |  |  |
| PFOS | No | 1898 | 0.16 | 0.4746 |  | 4.5701 (4.4522, 4.6912) |  |  |  |  |
|  | Yes | 39 | 0.00 |  |  | 4.2734 (3.6093, 5.0597) |  |  |  |  |

^*^ 95% CI: the 95% confidence intervals for the geometric mean were corrected for multiple comparisons using Bonferroni correction

^**^ The analysis is based on the non-parametric Wilcoxon test and Kaplan-Meier estimates of the median. Confidence intervals were calculated using the Greenwood variance estimate.

++ Caution should be taken since two groups have more than 50% observations undetected.

# Table S8: Results for MIREC persistent organic pollutants in maternal blood by maternal education (µg/L)

| **Contaminant** | **Group** | **N** | **%<LOD** | **Unadjusted** | | | **lipid as a covariate** | | | |
| --- | --- | --- | --- | --- | --- | --- | --- | --- | --- | --- |
|  |  |  |  | **P-Value** | **GM* (95% CI)** | | **P-Value for lipids** | **P-Value** | **GM* (95% CI)** | |
| Beta-HCH++ | High school or less | 171 | 53.80 | <.0001 | A | 0.0077 (0.0057, 0.0105) | <.0001 | <.0001 | A | 0.0083 (0.0064, 0.0106) |
|  | Some college and college | 542 | 38.56 |  | B | 0.0118 (0.0105, 0.0133) |  |  | A | 0.0109 (0.0096, 0.0125) |
|  | Undergraduate Degree or higher | 1183 | 25.53 |  | C | 0.0170 (0.0156, 0.0187) |  |  | B | 0.0172 (0.0158, 0.0188) |
| DDE | High school or less | 173 | 4.05 | <.0001 | A | 0.2707 (0.2288, 0.3203) | <.0001 | <.0001 | A | 0.2735 (0.2364, 0.3164) |
|  | Some college and college | 559 | 1.07 |  | A | 0.2965 (0.2749, 0.3198) |  |  | A | 0.2951 (0.2722, 0.3199) |
|  | Undergraduate Degree or higher | 1201 | 0.58 |  | B | 0.3801 (0.3593, 0.4021) |  |  | B | 0.3805 (0.3601, 0.4020) |
| Oxychlordane | High school or less | 173 | 23.70 | <.0001 | A | 0.0082 (0.0073, 0.0094) | <.0001 | <.0001 | A | 0.0085 (0.0077, 0.0094) |
|  | Some college and college | 558 | 8.24 |  | B | 0.0115 (0.0108, 0.0121) |  |  | B | 0.0114 (0.0108, 0.0120) |
|  | Undergraduate Degree or higher | 1200 | 5.17 |  | C | 0.0134 (0.0129, 0.0139) |  |  | C | 0.0134 (0.0130, 0.0139) |
| PBDE47 | High school or less | 173 | 28.90 | 0.0067 | A | 0.0482 (0.0398, 0.0583) | <.0001 | 0.0069 | A | 0.0480 (0.0395, 0.0584) |
|  | Some college and college | 557 | 30.34 |  | A | 0.0477 (0.0427, 0.0534) |  |  | A | 0.0474 (0.0425, 0.0529) |
|  | Undergraduate Degree or higher | 1196 | 36.87 |  | A | 0.0403 (0.0372, 0.0437) |  |  | A | 0.0405 (0.0375, 0.0438) |
| Arochlor1260 | High school or less | 173 | 13.87 | <.0001 | A | 0.2110 (0.1838, 0.2423) | <.0001 | <.0001 | A | 0.2136 (0.1885, 0.2421) |
|  | Some college and college | 559 | 2.86 |  | B | 0.3082 (0.2886, 0.3291) |  |  | B | 0.3064 (0.2861, 0.3282) |
|  | Undergraduate Degree or higher | 1201 | 1.00 |  | C | 0.4321 (0.4118, 0.4535) |  |  | C | 0.4326 (0.4129, 0.4533) |
| PCB118 | High school or less | 173 | 54.91 | <.0001 | A | 0.0087 (0.0072, 0.0105) | <.0001 | <.0001 | A | 0.0095 (0.0084, 0.0108) |
|  | Some college and college | 559 | 32.74 |  | B | 0.0127 (0.0119, 0.0135) |  |  | B | 0.0124 (0.0116, 0.0132) |
|  | Undergraduate Degree or higher | 1201 | 19.57 |  | C | 0.0165 (0.0158, 0.0172) |  |  | C | 0.0165 (0.0158, 0.0172) |
| PCB138 | High school or less | 173 | 24.28 | <.0001 | A | 0.0155 (0.0135, 0.0177) | <.0001 | <.0001 | A | 0.0157 (0.0139, 0.0178) |
|  | Some college and college | 559 | 8.59 |  | B | 0.0220 (0.0206, 0.0235) |  |  | B | 0.0218 (0.0204, 0.0234) |
|  | Undergraduate Degree or higher | 1201 | 3.75 |  | C | 0.0299 (0.0285, 0.0314) |  |  | C | 0.0300 (0.0286, 0.0314) |
| PCB153 | High school or less | 173 | 8.67 | <.0001 | A | 0.0253 (0.0220, 0.0290) | <.0001 | <.0001 | A | 0.0255 (0.0225, 0.0289) |
|  | Some college and college | 559 | 1.25 |  | B | 0.0371 (0.0347, 0.0397) |  |  | B | 0.0369 (0.0344, 0.0396) |
|  | Undergraduate Degree or higher | 1201 | 0.25 |  | C | 0.0530 (0.0504, 0.0556) |  |  | C | 0.0530 (0.0506, 0.0556) |
| PCB180 | High school or less | 173 | 28.32 | <.0001 | A | 0.0151 (0.0128, 0.0178) | <.0001 | <.0001 | A | 0.0154 (0.0133, 0.0179) |
|  | Some college and college | 559 | 10.38 |  | B | 0.0241 (0.0223, 0.0261) |  |  | B | 0.0240 (0.0222, 0.0259) |
|  | Undergraduate Degree or higher | 1201 | 3.00 |  | C | 0.0363 (0.0344, 0.0383) |  |  | C | 0.0363 (0.0344, 0.0383) |
| Transnonachlor | High school or less | 173 | 39.31 | <.0001 | A | 0.0116 (0.0101, 0.0133) | <.0001 | <.0001 | A | 0.0120 (0.0108, 0.0134) |
|  | Some college and college | 558 | 19.53 |  | B | 0.0158 (0.0149, 0.0168) |  |  | B | 0.0157 (0.0148, 0.0167) |
|  | Undergraduate Degree or higher | 1201 | 10.66 |  | C | 0.0199 (0.0191, 0.0207) |  |  | C | 0.0200 (0.0192, 0.0208) |
| PFHxS | High school or less | 172 | 5.23 | 0.3833 |  | 1.0320 (0.8758, 1.2162) |  |  |  |  |
|  | Some college and college | 563 | 3.73 |  |  | 1.0669 (0.9827, 1.1582) |  |  |  |  |
|  | Undergraduate Degree or higher | 1203 | 4.16 |  |  | 1.0100 (0.9579, 1.0650) |  |  |  |  |
| PFOA | High school or less | 172 | 0.00 | 0.0309 | A | 1.4962 (1.3509, 1.6572) |  |  |  |  |
|  | Some college and college | 563 | 0.18 |  | A | 1.7103 (1.6174, 1.8086) |  |  |  |  |
|  | Undergraduate Degree or higher | 1203 | 0.17 |  | A | 1.6426 (1.5755, 1.7125) |  |  |  |  |
| PFOS | High school or less | 172 | 0.00 | 0.0005 | A | 3.8758 (3.4900, 4.3042) |  |  |  |  |
|  | Some college and college | 563 | 0.36 |  | B | 4.6099 (4.3509, 4.8844) |  |  |  |  |
|  | Undergraduate Degree or higher | 1203 | 0.08 |  | B | 4.6474 (4.4647, 4.8375) |  |  |  |  |

^*^ 95% CI: the 95% confidence intervals for the geometric mean were corrected for multiple comparisons using Bonferroni correction

++ Caution should be taken since one group has more than 50% observations undetected.

# Table S9: Results for MIREC persistent organic pollutants in maternal blood by whether using non-stick cooking vessels

| Contaminant | Group | N | %<LOD | Unadjusted | | |  |  |  |  |
| --- | --- | --- | --- | --- | --- | --- | --- | --- | --- | --- |
|  |  |  |  | P-value | GM* (95% CI) | |  |  |  |  |
| PFHxS | No | 191 | 6.81 | 0.0818 |  | 0.9320 (0.8244, 1.0538) |  |  |  |  |
|  | Yes | 1745 | 3.84 |  |  | 1.0389 (1.0013, 1.0779) |  |  |  |  |
| PFOA | No | 191 | 0 | **0.0027** | A | 1.4580 (1.3354, 1.5919) |  |  |  |  |
|  | Yes | 1745 | 0.17 |  | B | **1.6672 (**1.6223, 1.7134) |  |  |  |  |
| PFOS | No | 191 | 0 | 0.3065 |  | 4.3794 (3.9991, 4.7958) |  |  |  |  |
|  | Yes | 1745 | 0.17 |  |  | 4.5820 (4.4604, 4.7069) |  |  |  |  |

* 95% CI: the 95% confidence intervals for the geometric mean were corrected for multiple comparisons using Bonferroni correction

# Table S10: Results for MIREC persistent organic pollutants in maternal blood by whether using non-stick cooking vessels in the microwave

| Contaminant | Group | N | %<LOD | Unadjusted | | |  |  |  |  |
| --- | --- | --- | --- | --- | --- | --- | --- | --- | --- | --- |
|  |  |  |  | P-value | GM* (95% CI) | |  |  |  |  |
| PFHxS | No | 1867 | 4.07 | 0.3477 |  | 1.0319 (0.9956, 1.0696) |  |  |  |  |
|  | Yes | 67 | 5.97 |  |  | 0.9367 (0.7556, 1.1612) |  |  |  |  |
| PFOA | No | 1867 | 0.16 | 0.4749 |  | 1.6495 (1.6061, 1.6942) |  |  |  |  |
|  | Yes | 67 | 0.00 |  |  | 1.5657 (1.3679, 1.7920) |  |  |  |  |
| PFOS | No | 1867 | 0.16 | 0.2708 |  | 4.5731 (4.4538, 4.6955) |  |  |  |  |
|  | Yes | 67 | 0.00 |  |  | 4.2239 (3.7395, 4.7710) |  |  |  |  |

^*^ 95% CI: the 95% confidence intervals for the geometric mean were corrected for multiple comparisons using Bonferroni correction

# Table S11: Results for MIREC persistent organic pollutants in maternal blood by year of collection

| Contaminant | group | N | %<LOD | Unadjusted | | | Lipid as a covariate | | | |
| --- | --- | --- | --- | --- | --- | --- | --- | --- | --- | --- |
|  |  |  |  | P-Value | GM* (95% CI) | | P-Value for lipids | P-Value | GM (95% CI) | |
| DDE | 2008 | 149 | 0.67 | <0.0001 | A | 0.3654 (0.3137, 0.4257) | 0.0018*** | |  |  |
|  | 2009 | 745 | 0.81 |  | A | 0.3682 (0.3395, 0.3993) |  |  |  |  |
|  | 2010 | 946 | 1.16 |  | A | 0.3335 (0.3108, 0.3578) |  |  |  |  |
|  | 2011 | 95 | 2.11 |  | B | 0.2360 (0.1992, 0.2797) |  |  |  |  |
| Oxychlordane | 2008 | 149 | 4.70 | <0.0001 | A | 0.0132 (0.0117, 0.0148) | <0.0001 | <0.0001 | A | 0.0135 (0.0120, 0.0152) |
|  | 2009 | 745 | 5.23 |  | A | 0.0133 (0.0126, 0.0141) |  |  | A | 0.0132 (0.0125, 0.0139) |
|  | 2010 | 945 | 9.21 |  | A | 0.0117 (0.0111, 0.0123) |  |  | A | 0.0118 (0.0112, 0.0123) |
|  | 2011 | 94 | 19.15 |  | B | 0.0092 (0.0077, 0.0109) |  |  | B | 0.0095 (0.0081, 0.0110) |
| PBDE47 | 2008 | 149 | 28.86 | 0.0031 | A | 0.0508 (0.0394, 0.0653) | 0.0019*** | |  |  |
|  | 2009 | 745 | 29.26 |  | A | 0.0472 (0.0426, 0.0522) |  |  |  |  |
|  | 2010 | 939 | 38.76 |  | A | 0.0393 (0.0355, 0.0435) |  |  |  |  |
|  | 2011 | 95 | 37.89 |  | A | 0.0382 (0.0264, 0.0552) |  |  |  |  |
| PCB | 2008 | 149 | 0.00 | <0.0001 | A | 0.4156 (0.3626, 0.4762) | <0.0001 | <0.0001 | A | 0.4238 (0.3637, 0.4939) |
|  | 2009 | 745 | 2.15 |  | A | 0.3879 (0.3616, 0.4163) |  |  | A | 0.3836 (0.3581, 0.4108) |
|  | 2010 | 946 | 3.28 |  | A | 0.3576 (0.3359, 0.3807) |  |  | A | 0.3591 (0.3379, 0.3817) |
|  | 2011 | 95 | 5.26 |  | B | 0.2599 (0.2168, 0.3117) |  |  | B | 0.2638 (0.2176, 0.3197) |
| PCB118 | 2008 | 149 | 18.12 | <0.0001 | A | 0.0172 (0.0151, 0.0197) | <0.0001 | <0.0001 | A | 0.0176 (0.0153, 0.0202) |
|  | 2009 | 745 | 24.83 |  | AB | 0.0149 (0.0139, 0.0159) |  |  | AB | 0.0146 (0.0138, 0.0156) |
|  | 2010 | 946 | 27.91 |  | B | 0.0142 (0.0133, 0.0150) |  |  | B | 0.0143 (0.0135, 0.0151) |
|  | 2011 | 95 | 41.05 |  | BC | 0.0114 (0.0095, 0.0136) |  |  | C | 0.0113 (0.0094, 0.0135) |
| PCB138 | 2008 | 149 | 2.01 | <0.0001 | A | 0.0294 (0.0258, 0.0336) | <0.0001 | <0.0001 | A | 0.0300 (0.0259, 0.0348) |
|  | 2009 | 745 | 5.50 |  | A | 0.0273 (0.0255, 0.0292) |  |  | A | 0.0270 (0.0252, 0.0288) |
|  | 2010 | 946 | 7.82 |  | A | 0.0251 (0.0236, 0.0267) |  |  | A | 0.0252 (0.0238, 0.0268) |
|  | 2011 | 95 | 18.95 |  | B | 0.0179 (0.0148, 0.0216) |  |  | B | 0.0181 (0.0150, 0.0219) |
| PCB153 | 2008 | 149 | 0.00 | <0.0001 | A | 0.0501 (0.0435, 0.0577) | <0.0001 | <0.0001 | A | 0.0510 (0.0436, 0.0597) |
|  | 2009 | 745 | 0.54 |  | A | 0.0472 (0.0439, 0.0507) |  |  | A | 0.0467 (0.0435, 0.0501) |
|  | 2010 | 946 | 1.90 |  | A | 0.0436 (0.0409, 0.0465) |  |  | A | 0.0438 (0.0411, 0.0466) |
|  | 2011 | 95 | 3.16 |  | B | 0.0315 (0.0261, 0.0380) |  |  | B | 0.0320 (0.0263, 0.0389) |
| PCB180 | 2008 | 149 | 2.68 | <0.0001 | A | 0.0355 (0.0303, 0.0416) | <0.0001 | <0.0001 | A | 0.0360 (0.0303, 0.0429) |
|  | 2009 | 745 | 6.44 |  | A | 0.0318 (0.0293, 0.0344) |  |  | A | 0.0315 (0.0291, 0.0340) |
|  | 2010 | 946 | 7.82 |  | A | 0.0288 (0.0268, 0.0309) |  |  | A | 0.0289 (0.0270, 0.0310) |
|  | 2011 | 95 | 17.89 |  | B | 0.0201 (0.0160, 0.0254) |  |  | B | 0.0204 (0.0164, 0.0255) |
| Transnonachlor | 2008 | 149 | 10.74 | <0.0001 | AB | 0.0198 (0.0175, 0.0225) | <0.0001 | <0.0001 | A | 0.0203 (0.0179, 0.0231) |
|  | 2009 | 745 | 12.48 |  | A | 0.0191 (0.0180, 0.0202) |  |  | A | 0.0188 (0.0178, 0.0199) |
|  | 2010 | 945 | 17.67 |  | B | 0.0171 (0.0162, 0.0180) |  |  | A | 0.0172 (0.0163, 0.0181) |
|  | 2011 | 95 | 32.63 |  | C | 0.0131 (0.0110, 0.0156) |  |  | B | 0.0133 (0.0113, 0.0157) |
| PFHxS | 2008 | 149 | 7.38 | 0.0476 | A | 0.8799 (0.7503, 1.0319) |  |  |  |  |
|  | 2009 | 753 | 5.31 |  | A | 1.0149 (0.9360, 1.1005) |  |  |  |  |
|  | 2010 | 943 | 2.86 |  | A | 1.0651 (0.9976, 1.1373) |  |  |  |  |
|  | 2011 | 95 | 2.11 |  | A | 1.0442 (0.8446, 1.2909) |  |  |  |  |
| PFOA | 2008 | 149 | 0.00 | 0.0030 | A | 1.6719 (1.4716, 1.8995) |  |  |  |  |
|  | 2009 | 753 | 0.00 |  | A | 1.7182 (1.6269, 1.8146) |  |  |  |  |
|  | 2010 | 943 | 0.32 |  | A | 1.6170 (1.5368, 1.7015) |  |  |  |  |
|  | 2011 | 95 | 0.00 |  | A | 1.3754 (1.1562, 1.6362) |  |  |  |  |
| PFOS | 2008 | 149 | 0.00 | <0.0001 | A | 5.3867 (4.7413, 6.1198) |  |  |  |  |
|  | 2009 | 753 | 0.00 |  | A | 4.8920 (4.6286, 5.1704) |  |  |  |  |
|  | 2010 | 943 | 0.32 |  | B | 4.3026 (4.0987, 4.5166) |  |  |  |  |
|  | 2011 | 95 | 0.00 |  | B | 3.6449 (3.0959, 4.2913) |  |  |  |  |

^*^ 95% CI: the 95% confidence intervals for the geometric mean were corrected for multiple comparisons using Bonferroni correction

^**^ The analysis is based on the non-parametric Wilcoxon test and Kaplan-Meier estimates of the median. Confidence intervals were calculated using the Greenwood variance estimate.

***Interaction between lipids and year of collection was significant

# Table S12: Results for MIREC persistent organic pollutants in maternal blood by intake of bacon

| Contaminant | bacon | N | %<LOD | Unadjusted | | | Lipid as a covariate | | | |
| --- | --- | --- | --- | --- | --- | --- | --- | --- | --- | --- |
|  |  |  |  | P-Value | GM* (95% CI) | | P-value for lipids | P-Value | GM (95% CI) | |
| Beta-HCH | never | 1090 | 31.01 | 0.2695** |  | 0.0140 (0.0132, 0.0148) | <0.0001 | **0.0231** | A | 0.0152 (0.0138, 0.0166) |
|  | 1 time a week | 475 | 33.47 |  |  | 0.0130 (0.0116, 0.0144) |  |  | A | 0.0129 (0.0112, 0.0149) |
|  | 2 times a week | 328 | 32.93 |  |  | 0.0130 (0.0117, 0.0143) |  |  | A | 0.0129 (0.0109, 0.0153) |
| DDE | never | 1104 | 0.82 | 0.1084 |  | 0.3534 (0.3328, 0.3754) | 0.0437*** | |  |  |
|  | 1 time a week | 489 | 1.23 |  |  | 0.3227 (0.2964, 0.3513) |  |  |  |  |
|  | 2 times a week | 336 | 1.49 |  |  | 0.3365 (0.3035, 0.3730) |  |  |  |  |
| Oxychlordane | never | 1103 | 7.52 | 0.7458 |  | 0.0124 (0.0119, 0.0129) | <0.0001 | 0.5965 |  | 0.0124 (0.0119, 0.0129) |
|  | 1 time a week | 489 | 9.00 |  |  | 0.0121 (0.0113, 0.0129) |  |  |  | 0.0121 (0.0114, 0.0128) |
|  | 2 times a week | 335 | 7.16 |  |  | 0.0122 (0.0113, 0.0131) |  |  |  | 0.0122 (0.0113, 0.0131) |
| PBDE47 | never | 1098 | 34.97 | 0.2671 |  | 0.0423 (0.0390, 0.0459) | 0.0067*** | |  |  |
|  | 1 time a week | 488 | 31.56 |  |  | 0.0462 (0.0409, 0.0521) |  |  |  |  |
|  | 2 times a week | 336 | 35.71 |  |  | 0.0413 (0.0355, 0.0480) |  |  |  |  |
| PCB | never | 1104 | 2.63 | 0.2671 |  | 0.3750 (0.3555, 0.3955) | <0.0001 | 0.2025 |  | 0.3758 (0.3570, 0.3956) |
|  | 1 time a week | 489 | 2.86 |  |  | 0.3525 (0.3262, 0.3810) |  |  |  | 0.3514 (0.3253, 0.3797) |
|  | 2 times a week | 336 | 2.68 |  |  | 0.3622 (0.3311, 0.3961) |  |  |  | 0.3609 (0.3288, 0.3962) |
| PCB118 | never | 1104 | 25.36 | 0.0622** |  | 0.0140 (0.0133, 0.0147) | <0.0001 | 0.0144 | A | 0.0150 (0.0143, 0.0157) |
|  | 1 time a week | 489 | 28.83 |  |  | 0.0140 (0.0129, 0.0151) |  |  | A | 0.0141 (0.0131, 0.0152) |
|  | 2 times a week | 336 | 27.98 |  |  | 0.0140 (0.0127, 0.0153) |  |  | A | 0.0134 (0.0123, 0.0146) |
| PCB138 | never | 1104 | 6.88 | 0.3010 |  | 0.0263 (0.0249, 0.0277) | <0.0001 | 0.2245 |  | 0.0264 (0.0251, 0.0277) |
|  | 1 time a week | 489 | 8.59 |  |  | 0.0248 (0.0230, 0.0268) |  |  |  | 0.0247 (0.0229, 0.0267) |
|  | 2 times a week | 336 | 5.36 |  |  | 0.0255 (0.0234, 0.0278) |  |  |  | 0.0254 (0.0232, 0.0278) |
| PCB153 | never | 1104 | 1.54 | 0.2671 |  | 0.0456 (0.0432, 0.0482) | <0.0001 | 0.2085 |  | 0.0457 (0.0434, 0.0482) |
|  | 1 time a week | 489 | 1.23 |  |  | 0.0428 (0.0396, 0.0463) |  |  |  | 0.0427 (0.0395, 0.0462) |
|  | 2 times a week | 336 | 0.60 |  |  | 0.0441 (0.0402, 0.0483) |  |  |  | 0.0440 (0.0400, 0.0484) |
| PCB170 | never | 1104 | 46.38 | 0.4945 |  | 0.0104 (0.0096, 0.0112) | <0.0001 | 0.4479 |  | 0.0105 (0.0098, 0.0113) |
|  | 1 time a week | 489 | 48.47 |  |  | 0.0101 (0.0091, 0.0113) |  |  |  | 0.0098 (0.0088, 0.0109) |
|  | 2 times a week | 336 | 46.43 |  |  | 0.0104 (0.0090, 0.0119) |  |  |  | 0.0103 (0.0091, 0.0117) |
| PCB180 | never | 1104 | 7.16 | 0.3171 |  | 0.0304 (0.0286, 0.0323) | <0.0001 | 0.2712 |  | 0.0305 (0.0288, 0.0323) |
|  | 1 time a week | 489 | 8.18 |  |  | 0.0285 (0.0261, 0.0311) |  |  |  | 0.0284 (0.0260, 0.0310) |
|  | 2 times a week | 336 | 7.14 |  |  | 0.0298 (0.0268, 0.0331) |  |  |  | 0.0297 (0.0267, 0.0331) |
| Transnonachlor | never | 1104 | 16.58 | 0.9227 |  | 0.0178 (0.0170, 0.0186) | <0.0001 | 0.8461 |  | 0.0179 (0.0171, 0.0187) |
|  | 1 time a week | 488 | 15.78 |  |  | 0.0178 (0.0166, 0.0191) |  |  |  | 0.0178 (0.0167, 0.0190) |
|  | 2 times a week | 336 | 13.99 |  |  | 0.0176 (0.0163, 0.0190) |  |  |  | 0.0175 (0.0162, 0.0189) |
| PFHxS | never | 1105 | 4.34 | 0.1028 |  | 1.0171 (0.9605, 1.0771) |  |  |  |  |
|  | 1 time a week | 492 | 4.88 |  |  | 0.9983 (0.9159, 1.0881) |  |  |  |  |
|  | 2 times a week | 337 | 2.37 |  |  | 1.1172 (1.0086, 1.2374) |  |  |  |  |
| PFOA | never | 1105 | 0.09 | 0.0824 |  | 1.6449 (1.5770, 1.7158) |  |  |  |  |
|  | 1 time a week | 492 | 0.41 |  |  | 1.5850 (1.4879, 1.6884) |  |  |  |  |
|  | 2 times a week | 337 | 0.00 |  |  | 1.7391 (1.6097, 1.8789) |  |  |  |  |
| PFOS | never | 1105 | 0.18 | 0.5410 |  | 4.5123 (4.3227, 4.7103) |  |  |  |  |
|  | 1 time a week | 492 | 0.20 |  |  | 4.5899 (4.3201, 4.8765) |  |  |  |  |
|  | 2 times a week | 337 | 0.00 |  |  | 4.6913 (4.3654, 5.0415) |  |  |  |  |

^*^ 95% CI: the 95% confidence intervals for the geometric mean were corrected for multiple comparisons using Bonferroni correction

^**^ The analysis is based on the non-parametric Wilcoxon test and Kaplan-Meier estimates of the median. Confidence intervals were calculated using the Greenwood variance estimate.

***Interaction between lipids and intake of bacon was significant

# Table S13: Results for MIREC persistent organic pollutants in maternal blood by intake of fish

| Contaminant | Group | N | %<LOD | Unadjusted | | | Lipid as a covariate | | | |
| --- | --- | --- | --- | --- | --- | --- | --- | --- | --- | --- |
|  |  |  |  | P-value | GM* (95% CI) | | P-value for lipids | P-value | GM (95% CI) | |
| Beta-HCH | never | 1101 | 35.06 | <0.0001 | A | 0.0128 (0.0116, 0.0141) | <0.0001 | <0.0001 | A | 0.0126 (0.0115, 0.0138) |
|  | 1 time a week | 456 | 26.97 |  | B | 0.0163 (0.0142, 0.0186) |  |  | B | 0.0160 (0.0139, 0.0183) |
|  | 2 times a week | 336 | 28.57 |  | B | 0.0169 (0.0140, 0.0203) |  |  | B | 0.0176 (0.0150, 0.0207) |
| DDE | never | 1120 | 1.16 | 0.0004 | A | 0.3225 (0.3046, 0.3414) | <0.0001 | 0.0001 | A | 0.3215 (0.3035, 0.3406) |
|  | 1 time a week | 465 | 0.43 |  | AB | 0.3615 (0.3314, 0.3943) |  |  | AB | 0.3612 (0.3304, 0.3950) |
|  | 2 times a week | 344 | 1.45 |  | B | 0.3867 (0.3443, 0.4344) |  |  | B | 0.3910 (0.3524, 0.4338) |
| Oxychlordane | never | 1118 | 9.48 | <0.0001 | A | 0.0117 (0.0112, 0.0122) | <0.0001 | <0.0001 | A | 0.0116 (0.0112, 0.0121) |
|  | 1 time a week | 465 | 6.02 |  | B | 0.0132 (0.0124, 0.0141) |  |  | B | 0.0132 (0.0124, 0.0140) |
|  | 2 times a week | 344 | 4.94 |  | B | 0.0131 (0.0122, 0.0141) |  |  | B | 0.0133 (0.0124, 0.0143) |
| PBDE47 | never | 1119 | 31.99 | 0.0034 | A | 0.0462 (0.0426, 0.0501) | <0.0001 | 0.0054 | A | 0.0461 (0.0426, 0.0499) |
|  | 1 time a week | 463 | 36.93 |  | A | 0.0399 (0.0352, 0.0451) |  |  | A | 0.0394 (0.0349, 0.0446) |
|  | 2 times a week | 340 | 37.94 |  | A | 0.0381 (0.0327, 0.0445) |  |  | A | 0.0389 (0.0337, 0.0449) |
| PCB | never | 1120 | 3.75 | <0.0001 | A | 0.3262 (0.3102, 0.3431) | <0.0001 | <0.0001 | A | 0.3251 (0.3093, 0.3418) |
|  | 1 time a week | 465 | 1.08 |  | B | 0.4045 (0.3745, 0.4369) |  |  | B | 0.4042 (0.3741, 0.4367) |
|  | 2 times a week | 344 | 1.45 |  | B | 0.4717 (0.4284, 0.5194) |  |  | B | 0.4771 (0.4360, 0.5221) |
| PCB118 | never | 1120 | 30.63 | <0.0001 | A | 0.0134 (0.0128, 0.0141) | <0.0001 | <0.0001 | A | 0.0133 (0.0127, 0.0139) |
|  | 1 time a week | 465 | 23.87 |  | B | 0.0153 (0.0142, 0.0165) |  |  | B | 0.0153 (0.0143, 0.0164) |
|  | 2 times a week | 344 | 17.73 |  | B | 0.0173 (0.0159, 0.0189) |  |  | B | 0.0176 (0.0162, 0.0191) |
| PCB138 | never | 1120 | 8.84 | <0.0001 | A | 0.0232 (0.0221, 0.0244) | <0.0001 | <0.0001 | A | 0.0231 (0.0220, 0.0243) |
|  | 1 time a week | 465 | 4.95 |  | B | 0.0283 (0.0262, 0.0305) |  |  | B | 0.0282 (0.0262, 0.0304) |
|  | 2 times a week | 344 | 4.07 |  | B | 0.0319 (0.0291, 0.0351) |  |  | B | 0.0324 (0.0296, 0.0353) |
| PCB153 | never | 1120 | 1.79 | <0.0001 | A | 0.0394 (0.0374, 0.0415) | <0.0001 | <0.0001 | A | 0.0393 (0.0373, 0.0413) |
|  | 1 time a week | 465 | 0.65 |  | B | 0.0493 (0.0456, 0.0533) |  |  | B | 0.0493 (0.0455, 0.0533) |
|  | 2 times a week | 344 | 0.58 |  | B | 0.0585 (0.0530, 0.0645) |  |  | C | 0.0591 (0.0539, 0.0648) |
| PCB180 | never | 1120 | 9.55 | <0.0001 | A | 0.0260 (0.0246, 0.0275) | <0.0001 | <0.0001 | A | 0.0259 (0.0245, 0.0275) |
|  | 1 time a week | 465 | 4.52 |  | B | 0.0331 (0.0304, 0.0361) |  |  | B | 0.0331 (0.0303, 0.0361) |
|  | 2 times a week | 344 | 4.36 |  | B | 0.0404 (0.0362, 0.0451) |  |  | C | 0.0409 (0.0369, 0.0453) |
| Transnonachlor | never | 1120 | 19.29 | <0.0001 | A | 0.0163 (0.0156, 0.0171) | <0.0001 | <0.0001 | A | 0.0163 (0.0156, 0.0170) |
|  | 1 time a week | 465 | 11.40 |  | B | 0.0198 (0.0185, 0.0211) |  |  | B | 0.0197 (0.0185, 0.0211) |
|  | 2 times a week | 343 | 11.08 |  | B | 0.0204 (0.0188, 0.0221) |  |  | B | 0.0207 (0.0192, 0.0223) |
| PFHxS | never | 1123 | 4.19 | 0.0536 |  | 1.0674 (1.0076, 1.1307) |  |  |  |  |
|  | 1 time a week | 466 | 3.86 |  |  | 0.9728 (0.8950, 1.0573) |  |  |  |  |
|  | 2 times a week | 345 | 4.35 |  |  | 0.9855 (0.8882, 1.0935) |  |  |  |  |
| PFOA | never | 1123 | 0.18 | 0.0770 |  | 1.6834 (1.6149, 1.7549) |  |  |  |  |
|  | 1 time a week | 466 | 0.00 |  |  | 1.5651 (1.4637, 1.6735) |  |  |  |  |
|  | 2 times a week | 345 | 0.29 |  |  | 1.6341 (1.5166, 1.7607) |  |  |  |  |
| PFOS | never | 1123 | 0.09 | 0.8598 |  | 4.5363 (4.3545, 4.7258) |  |  |  |  |
|  | 1 time a week | 466 | 0.21 |  |  | 4.5848 (4.2856, 4.9048) |  |  |  |  |
|  | 2 times a week | 345 | 0.29 |  |  | 4.6197 (4.2956, 4.9681) |  |  |  |  |

^*^ 95% CI: the 95% confidence intervals for the geometric mean were corrected for multiple comparisons using Bonferroni correction

# Table S14: Results for MIREC persistent organic pollutants in maternal blood by intake of Hamburger

| Contaminant | Group | N | %<LOD | Unadjusted | | | Lipid as a covariate | | | |
| --- | --- | --- | --- | --- | --- | --- | --- | --- | --- | --- |
|  |  |  |  | P-value | GM* (95% CI) | | P-value for lipids | P-value | GM (95% CI) | |
| Beta-HCH | never | 887 | 30.33 | <0.0001 | A | 0.0154 (0.0134, 0.0176) | <0.0001 | <0.0001 | A | **0.0162** (0.0145, 0.0181) |
|  | 1 time a week | 548 | 30.11 |  | A | 0.0149 (0.0132, 0.0169) |  |  | AB | 0.0141 (0.0122, 0.0162) |
|  | 2 times a week | 297 | 37.04 |  | A | 0.0122 (0.0103, 0.0144) |  |  | B | 0.0112 (0.0092, 0.0137) |
|  | >2 time a week | 161 | 37.89 |  | A | 0.0119 (0.0096, 0.0149) |  |  | B | 0.0105 (0.0080, 0.0137) |
| DDE | never | 902 | 0.89 | <0.0001 | A | 0.3755 (0.3467, 0.4067) | <0.0001 | <0.0001 | A | **0.3765 (**0.3508, 0.4039) |
|  | 1 time a week | 561 | 1.25 |  | AB | 0.3343 (0.3087, 0.3620) |  |  | AB | 0.3340 (0.3054, 0.3652) |
|  | 2 times a week | 300 | 1.33 |  | AB | 0.3005 (0.2691, 0.3357) |  |  | B | 0.3003 (0.2657, 0.3393) |
|  | >2 time a week | 166 | 0.60 |  | B | 0.2851 (0.2478, 0.3279) |  |  | B | 0.2820 (0.2393, 0.3324) |
| Oxychlordane | never | 902 | 7.32 | 0.0542** |  | 0.0130 (0.0123, 0.0137) | <0.0001 | **0.0137** | A | 0.0126 (0.0120, 0.0132) |
|  | 1 time a week | 561 | 7.49 |  |  | 0.0130 (0.0120, 0.0140) |  |  | A | 0.0125 (0.0118, 0.0133) |
|  | 2 times a week | 298 | 7.38 |  |  | 0.0120 (0.0106, 0.0134) |  |  | A | 0.0117 (0.0107, 0.0127) |
|  | >2 time a week | 166 | 12.65 |  |  | 0.0110 (0.0098, 0.0122) |  |  | A | 0.0111 (0.0099, 0.0124) |
| PBDE47 | never | 899 | 36.93 | 0.1369 |  | 0.0403 (0.0364, 0.0447) | <0.0001 | 0.1414 |  | 0.0406 (0.0368, 0.0447) |
|  | 1 time a week | 557 | 30.70 |  |  | 0.0467 (0.0415, 0.0525) |  |  |  | 0.0460 (0.0408, 0.0520) |
|  | 2 times a week | 300 | 32.67 |  |  | 0.0446 (0.0373, 0.0534) |  |  |  | 0.0452 (0.0383, 0.0533) |
|  | >2 time a week | 166 | 34.34 |  |  | 0.0438 (0.0345, 0.0556) |  |  |  | 0.0436 (0.0349, 0.0544) |
| PCB | never | 902 | 2.22 | <0.0001 | A | 0.3923 (0.3671, 0.4192) | <0.0001 | <0.0001 | A | 0.3932 (0.3696, 0.4185) |
|  | 1 time a week | 561 | 1.96 |  | AB | 0.3771 (0.3489, 0.4076) |  |  | AB | 0.3769 (0.3484, 0.4078) |
|  | 2 times a week | 300 | 3.67 |  | B | 0.3215 (0.2896, 0.3569) |  |  | B | 0.3212 (0.2883, 0.3577) |
|  | >2 time a week | 166 | 6.02 |  | BC | 0.2962 (0.2604, 0.3370) |  |  | BC | 0.2919 (0.2524, 0.3375) |
| PCB118 | never | 902 | 25.39 | <0.0001 | A | 0.0152 (0.0142, 0.0161) | <0.0001 | <0.0001 | A | 0.0153 (0.0145, 0.0162) |
|  | 1 time a week | 561 | 24.42 |  | A | 0.0149 (0.0138, 0.0160) |  |  | AB | 0.0149 (0.0139, 0.0160) |
|  | 2 times a week | 300 | 31.67 |  | A | 0.0130 (0.0118, 0.0143) |  |  | B | 0.0128 (0.0116, 0.0141) |
|  | >2 time a week | 166 | 32.53 |  | A | 0.0127 (0.0113, 0.0143) |  |  | BC | 0.0122 (0.0106, 0.0139) |
| PCB138 | never | 902 | 5.76 | <0.0001 | A | 0.0274 (0.0257, 0.0292) | <0.0001 | <0.0001 | A | 0.0275 (0.0258, 0.0292) |
|  | 1 time a week | 561 | 7.31 |  | AB | 0.0264 (0.0244, 0.0286) |  |  | AB | 0.0264 (0.0244, 0.0285) |
|  | 2 times a week | 300 | 9.33 |  | AB | 0.0227 (0.0206, 0.0252) |  |  | B | 0.0227 (0.0204, 0.0252) |
|  | >2 time a week | 166 | 9.04 |  | B | 0.0217 (0.0192, 0.0245) |  |  | B | 0.0213 (0.0184, 0.0245) |
| PCB153 | never | 902 | 1.33 | <0.0001 | A | 0.0478 (0.0447, 0.0512) | <0.0001 | <0.0001 | A | 0.0480 (0.0450, 0.0511) |
|  | 1 time a week | 561 | 1.07 |  | AB | 0.0458 (0.0423, 0.0495) |  |  | AB | 0.0458 (0.0422, 0.0496) |
|  | 2 times a week | 300 | 1.33 |  | B | 0.0391 (0.0352, 0.0435) |  |  | B | 0.0391 (0.0350, 0.0437) |
|  | >2 time a week | 166 | 1.81 |  | BC | 0.0357 (0.0314, 0.0406) |  |  | BC | 0.0353 (0.0304, 0.0409) |
| PCB180 | never | 902 | 6.43 | <0.0001 | A | 0.0322 (0.0298, 0.0347) | <0.0001 | <0.0001 | A | 0.0323 (0.0301, 0.0347) |
|  | 1 time a week | 561 | 6.95 |  | AB | 0.0304 (0.0279, 0.0332) |  |  | AB | 0.0303 (0.0277, 0.0332) |
|  | 2 times a week | 300 | 9.33 |  | B | 0.0263 (0.0234, 0.0296) |  |  | B | 0.0262 (0.0232, 0.0297) |
|  | >2 time a week | 166 | 10.84 |  | BC | 0.0232 (0.0201, 0.0269) |  |  | BC | 0.0228 (0.0193, 0.0269) |
| Transnonachlor | never | 902 | 15.96 | 0.0200 | A | 0.0183 (0.0173, 0.0194) | <0.0001 | 0.0059 | A | 0.0184 (0.0175, 0.0194) |
|  | 1 time a week | 560 | 14.46 |  | A | 0.0180 (0.0169, 0.0192) |  |  | A | 0.0180 (0.0168, 0.0192) |
|  | 2 times a week | 300 | 17.00 |  | A | 0.0168 (0.0152, 0.0185) |  |  | A | 0.0168 (0.0154, 0.0184) |
|  | >2 time a week | 166 | 18.67 |  | A | 0.0160 (0.0141, 0.0182) |  |  | A | 0.0158 (0.0140, 0.0179) |
| PFHxS | never | 906 | 5.52 | 0.1841 |  | 0.9867 (0.9193, 1.0590) |  |  |  |  |
|  | 1 time a week | 563 | 3.02 |  |  | 1.0574 (0.9686, 1.1544) |  |  |  |  |
|  | 2 times a week | 300 | 3.00 |  |  | 1.0818 (0.9662, 1.2111) |  |  |  |  |
|  | >2 time a week | 165 | 2.42 |  |  | 1.0769 (0.9073, 1.2782) |  |  |  |  |
| PFOA | never | 906 | 0.11 | 0.7299 |  | 1.6587 (1.5749, 1.7469) |  |  |  |  |
|  | 1 time a week | 563 | 0.36 |  |  | 1.6266 (1.5223, 1.7382) |  |  |  |  |
|  | 2 times a week | 300 | 0.00 |  |  | 1.6742 (1.5350, 1.8260) |  |  |  |  |
|  | >2 time a week | 165 | 0.00 |  |  | 1.5858 (1.4113, 1.7818) |  |  |  |  |
| PFOS | never | 906 | 0.11 | 0.9297 |  | 4.5574 (4.3235, 4.8039) |  |  |  |  |
|  | 1 time a week | 563 | 0.36 |  |  | 4.5706 (4.2888, 4.8709) |  |  |  |  |
|  | 2 times a week | 300 | 0.00 |  |  | 4.6247 (4.2576, 5.0234) |  |  |  |  |
|  | >2 time a week | 165 | 0.00 |  |  | 4.4550 (3.9760, 4.9917) |  |  |  |  |

^*^ 95% CI: the 95% confidence intervals for the geometric mean were corrected for multiple comparisons using Bonferroni correction

^**^ The analysis is based on the non-parametric Wilcoxon test and Kaplan-Meier estimates of the median. Confidence intervals were calculated using the Greenwood variance estimate.

# Table S15: Results for MIREC persistent organic pollutants in maternal blood by intake of pork

| Contaminant | Group | N | %<LOD | Unadjusted | | | Lipid as a covariate | | | |
| --- | --- | --- | --- | --- | --- | --- | --- | --- | --- | --- |
|  |  |  |  | P-value | GM* (95% CI) | | P-Value for lipids | P-Value | GM (95% CI) | |
| Beta-HCH | never | 1321 | 32.48 | 0.7003 |  | 0.0141 (0.0130, 0.0154) | <0.0001 | 0.6750 |  | 0.0140 (0.0128, 0.0152) |
|  | 1 time a week | 366 | 27.87 |  |  | 0.0149 (0.0129, 0.0172) |  |  |  | 0.0142 (0.0122, 0.0167) |
|  | 2 times a week | 206 | 35.92 |  |  | 0.0136 (0.0102, 0.0181) |  |  |  | 0.0152 (0.0123, 0.0188) |
| DDE | never | 1346 | 1.34 | 0.4520 |  | 0.3402 (0.3228, 0.3585) | <0.0001 | 0.4081 |  | 0.3403 (0.3228, 0.3587) |
|  | 1 time a week | 372 | 0.54 |  |  | 0.3378 (0.3075, 0.3711) |  |  |  | 0.3367 (0.3045, 0.3722) |
|  | 2 times a week | 211 | 0.00 |  |  | 0.3659 (0.3116, 0.4297) |  |  |  | 0.3669 (0.3212, 0.4192) |
| Oxychlordane | never | 1344 | 8.33 | 0.8441 |  | 0.0122 (0.0118, 0.0127) | <0.0001 | 0.9122 |  | 0.0122 (0.0118, 0.0127) |
|  | 1 time a week | 372 | 6.18 |  |  | 0.0125 (0.0117, 0.0134) |  |  |  | 0.0124 (0.0116, 0.0133) |
|  | 2 times a week | 211 | 7.58 |  |  | 0.0122 (0.0110, 0.0135) |  |  |  | 0.0123 (0.0112, 0.0134) |
| PBDE47 | never | 1342 | 34.72 | 0.8680 |  | 0.0427 (0.0396, 0.0460) | <0.0001 | 0.8343 |  | 0.0428 (0.0398, 0.0461) |
|  | 1 time a week | 371 | 33.15 |  |  | 0.0437 (0.0382, 0.0500) |  |  |  | 0.0430 (0.0375, 0.0492) |
|  | 2 times a week | 209 | 33.01 |  |  | 0.0444 (0.0367, 0.0537) |  |  |  | 0.0449 (0.0376, 0.0538) |
| PCB | never | 1346 | 2.67 | 0.1802 |  | 0.3691 (0.3519, 0.3872) | <0.0001 | 0.2012 |  | 0.3693 (0.3525, 0.3869) |
|  | 1 time a week | 372 | 2.15 |  |  | 0.3765 (0.3442, 0.4118) |  |  |  | 0.3751 (0.3434, 0.4099) |
|  | 2 times a week | 211 | 3.79 |  |  | 0.3377 (0.3026, 0.3768) |  |  |  | 0.3382 (0.3007, 0.3805) |
| PCB118 | never | 1346 | 26.82 | 0.0533 |  | 0.0146 (0.0140, 0.0153) | <0.0001 | 0.0612 |  | 0.0146 (0.0140, 0.0153) |
|  | 1 time a week | 372 | 24.73 |  |  | 0.0148 (0.0136, 0.0161) |  |  |  | 0.0147 (0.0135, 0.0159) |
|  | 2 times a week | 211 | 29.38 |  |  | 0.0133 (0.0120, 0.0147) |  |  |  | 0.0131 (0.0117, 0.0146) |
| PCB138 | never | 1346 | 7.28 | 0.2199 |  | 0.0259 (0.0247, 0.0272) | <0.0001 | 0.2465 |  | 0.0259 (0.0248, 0.0271) |
|  | 1 time a week | 372 | 5.91 |  |  | 0.0264 (0.0242, 0.0288) |  |  |  | 0.0263 (0.0241, 0.0286) |
|  | 2 times a week | 211 | 7.58 |  |  | 0.0239 (0.0216, 0.0266) |  |  |  | 0.0239 (0.0213, 0.0268) |
| PCB153 | never | 1346 | 1.34 | 0.2144 |  | 0.0449 (0.0427, 0.0471) | <0.0001 | 0.2377 |  | 0.0449 (0.0428, 0.0471) |
|  | 1 time a week | 372 | 1.34 |  |  | 0.0458 (0.0418, 0.0502) |  |  |  | 0.0456 (0.0417, 0.0500) |
|  | 2 times a week | 211 | 0.95 |  |  | 0.0412 (0.0368, 0.0460) |  |  |  | 0.0413 (0.0366, 0.0465) |
| PCB170++ | never | 1346 | 46.95 | 0.1324 |  | 0.0103 (0.0096, 0.0110) | <0.0001 | 0.1458 |  | 0.0104 (0.0097, 0.0111) |
|  | 1 time a week | 372 | 44.62 |  |  | 0.0109 (0.0096, 0.0123) |  |  |  | 0.0107 (0.0095, 0.0121) |
|  | 2 times a week | 211 | 50.71 |  |  | 0.0097 (0.0083, 0.0113) |  |  |  | 0.0091 (0.0077, 0.0108) |
| PCB180 | never | 1346 | 7.36 | 0.1063 |  | 0.0300 (0.0284, 0.0316) | <0.0001 | 0.1221 |  | 0.0300 (0.0284, 0.0316) |
|  | 1 time a week | 372 | 6.99 |  |  | 0.0311 (0.0281, 0.0343) |  |  |  | 0.0309 (0.0280, 0.0342) |
|  | 2 times a week | 211 | 8.53 |  |  | 0.0269 (0.0238, 0.0305) |  |  |  | 0.0269 (0.0235, 0.0308) |
| Transnonachlor | never | 1346 | 16.72 | 0.4913 |  | 0.0176 (0.0169, 0.0183) | <0.0001 | 0.5011 |  | 0.0176 (0.0169, 0.0183) |
|  | 1 time a week | 371 | 13.75 |  |  | 0.0183 (0.0171, 0.0197) |  |  |  | 0.0182 (0.0169, 0.0196) |
|  | 2 times a week | 211 | 14.69 |  |  | 0.0181 (0.0163, 0.0202) |  |  |  | 0.0183 (0.0166, 0.0201) |
| PFHxS | never | 1347 | 4.01 | 0.6255 |  | 1.0176 (0.9670, 1.0708) |  |  |  |  |
|  | 1 time a week | 376 | 4.79 |  |  | 1.0539 (0.9522, 1.1665) |  |  |  |  |
|  | 2 times a week | 211 | 3.79 |  |  | 1.0591 (0.9234, 1.2146) |  |  |  |  |
| PFOA | never | 1347 | 0.07 | 0.1066 |  | 1.6758 (1.6142, 1.7398) |  |  |  |  |
|  | 1 time a week | 376 | 0.53 |  |  | 1.5889 (1.4674, 1.7205) |  |  |  |  |
|  | 2 times a week | 211 | 0.00 |  |  | 1.5573 (1.4197, 1.7083) |  |  |  |  |
| PFOS | never | 1347 | 0.07 | 0.6390 |  | 4.5382 (4.3715, 4.7113) |  |  |  |  |
|  | 1 time a week | 376 | 0.53 |  |  | 4.6791 (4.3467, 5.0369) |  |  |  |  |
|  | 2 times a week | 211 | 0.00 |  |  | 4.5150 (4.0969, 4.9757) |  |  |  |  |

^*^ 95% CI: the 95% confidence intervals for the geometric mean were corrected for multiple comparisons using Bonferroni correction

++ Caution should be taken since one group has more than 50% observations undetected.

# Table S16: Results for MIREC persistent organic pollutants in maternal blood by intake of poultry

| Contaminant | Group | N | %<LOD | unadjusted | | | Lipid as a covariate | | | |
| --- | --- | --- | --- | --- | --- | --- | --- | --- | --- | --- |
|  |  |  |  | P-value | GM* (95% CI) | | P-Value for lipids | P-Value | GM (95% CI) | |
| Beta-HCH | never | 409 | 33.01 | 0.6197 |  | 0.0143 (0.0117, 0.0175) | <0.0001 | 0.3787 |  | 0.0154 (0.0131, 0.0182) |
|  | 1 time a week | 460 | 33.70 |  |  | 0.0137 (0.0116, 0.0161) |  |  |  | 0.0134 (0.0115, 0.0157) |
|  | 2 times a week | 492 | 28.46 |  |  | 0.0148 (0.0129, 0.0171) |  |  |  | 0.0143 (0.0123, 0.0166) |
|  | >2 time a week | 532 | 32.89 |  |  | 0.0141 (0.0122, 0.0163) |  |  |  | 0.0137 (0.0118, 0.0159) |
| DDE | never | 418 | 1.20 | 0.3319 |  | 0.3477 (0.3118, 0.3879) | <0.0001 | 0.2989 |  | 0.3509 (0.3161, 0.3895) |
|  | 1 time a week | 469 | 0.85 |  |  | 0.3576 (0.3236, 0.3951) |  |  |  | 0.3561 (0.3227, 0.3930) |
|  | 2 times a week | 501 | 1.00 |  |  | 0.3421 (0.3108, 0.3767) |  |  |  | 0.3417 (0.3107, 0.3759) |
|  | >2 time a week | 541 | 1.11 |  |  | 0.3261 (0.2981, 0.3567) |  |  |  | 0.3253 (0.2968, 0.3565) |
| Oxychlordane | never | 418 | 7.42 | 0.0551 |  | 0.0129 (0.0120, 0.0140) | <0.0001 | 0.0133 | A | 0.0131 (0.0122, 0.0141) |
|  | 1 time a week | 469 | 6.82 |  |  | 0.0124 (0.0116, 0.0133) |  |  | A | 0.0124 (0.0116, 0.0132) |
|  | 2 times a week | 499 | 8.22 |  |  | 0.0122 (0.0114, 0.0131) |  |  | A | 0.0122 (0.0114, 0.0130) |
|  | >2 time a week | 541 | 8.69 |  |  | 0.0117 (0.0110, 0.0125) |  |  | A | 0.0117 (0.0110, 0.0124) |
| PBDE47 | never | 418 | 32.54 | 0.7559 |  | 0.0447 (0.0389, 0.0514) | <0.0001 | 0.6854 |  | 0.0448 (0.0389, 0.0515) |
|  | 1 time a week | 466 | 30.90 |  |  | 0.0450 (0.0394, 0.0512) |  |  |  | 0.0442 (0.0387, 0.0504) |
|  | 2 times a week | 501 | 36.53 |  |  | 0.0414 (0.0361, 0.0475) |  |  |  | 0.0415 (0.0365, 0.0473) |
|  | >2 time a week | 537 | 36.31 |  |  | 0.0416 (0.0363, 0.0478) |  |  |  | 0.0423 (0.0373, 0.0480) |
| PCB | never | 418 | 2.63 | 0.0764** |  | 0.3600 (0.3280, 0.3920) | <0.0001 | 0.0284 | A | 0.3769 (0.3438, 0.4132) |
|  | 1 time a week | 469 | 1.71 |  |  | 0.3700 (0.3252, 0.4148) |  |  | A | 0.3914 (0.3588, 0.4268) |
|  | 2 times a week | 501 | 2.99 |  |  | 0.3500 (0.3103, 0.3897) |  |  | A | 0.3620 (0.3328, 0.3937) |
|  | >2 time a week | 541 | 3.33 |  |  | 0.3300 (0.3026, 0.3574) |  |  | A | 0.3439 (0.3172, 0.3729) |
| PCB118 | never | 418 | 26.79 | 0.0244 | A | 0.0148 (0.0135, 0.0163) | <0.0001 | 0.0139 | A | 0.0152 (0.0139, 0.0165) |
|  | 1 time a week | 469 | 23.45 |  | A | 0.0153 (0.0141, 0.0167) |  |  | A | 0.0152 (0.0141, 0.0165) |
|  | 2 times a week | 501 | 26.95 |  | A | 0.0143 (0.0132, 0.0154) |  |  | A | 0.0142 (0.0131, 0.0153) |
|  | >2 time a week | 541 | 29.21 |  | A | 0.0137 (0.0126, 0.0148) |  |  | A | 0.0136 (0.0126, 0.0146) |
| PCB138 | never | 418 | 6.22 | 0.0789 |  | 0.0261 (0.0237, 0.0286) | <0.0001 | 0.0677 |  | 0.0263 (0.0241, 0.0288) |
|  | 1 time a week | 469 | 5.97 |  |  | 0.0275 (0.0251, 0.0300) |  |  |  | 0.0273 (0.0251, 0.0298) |
|  | 2 times a week | 501 | 7.39 |  |  | 0.0254 (0.0234, 0.0276) |  |  |  | 0.0253 (0.0233, 0.0275) |
|  | >2 time a week | 541 | 8.32 |  |  | 0.0246 (0.0227, 0.0266) |  |  |  | 0.0245 (0.0226, 0.0265) |
| PCB153 | never | 418 | 1.91 | 0.0570** |  | 0.0420 (0.0379, 0.0461) | <0.0001 | 0.0238 | A | 0.0459 (0.0418, 0.0504) |
|  | 1 time a week | 469 | 0.85 |  |  | 0.0440 (0.0393, 0.0487) |  |  | A | 0.0477 (0.0436, 0.0521) |
|  | 2 times a week | 501 | 1.20 |  |  | 0.0430 (0.0382, 0.0478) |  |  | A | 0.0442 (0.0406, 0.0481) |
|  | >2 time a week | 541 | 1.29 |  |  | 0.0400 (0.0362, 0.0438) |  |  | A | 0.0416 (0.0383, 0.0452) |
| PCB170 | never | 418 | 48.56 | 0.1043 |  | 0.0099 (0.0085, 0.0115) | <0.0001 | 0.1121 |  | 0.0103 (0.0091, 0.0117) |
|  | 1 time a week | 469 | 42.86 |  |  | 0.0112 (0.0098, 0.0126) |  |  |  | 0.0111 (0.0098, 0.0125) |
|  | 2 times a week | 501 | 46.31 |  |  | 0.0105 (0.0093, 0.0119) |  |  |  | 0.0104 (0.0092, 0.0117) |
|  | >2 time a week | 541 | 49.72 |  |  | 0.0097 (0.0086, 0.0110) |  |  |  | 0.0095 (0.0085, 0.0107) |
| PCB180 | never | 418 | 6.46 | 0.0126 | A | 0.0309 (0.0277, 0.0344) | <0.0001 | 0.0099 | A | 0.0311 (0.0280, 0.0346) |
|  | 1 time a week | 469 | 5.54 |  | A | 0.0321 (0.0290, 0.0354) |  |  | A | 0.0320 (0.0289, 0.0353) |
|  | 2 times a week | 501 | 7.78 |  | A | 0.0298 (0.0270, 0.0328) |  |  | A | 0.0298 (0.0270, 0.0327) |
|  | >2 time a week | 541 | 9.43 |  | A | 0.0273 (0.0249, 0.0299) |  |  | A | 0.0272 (0.0248, 0.0298) |
| Transnonachlor | never | 418 | 16.51 | 0.1730 |  | 0.0180 (0.0166, 0.0196) | <0.0001 | 0.1209 |  | 0.0183 (0.0170, 0.0198) |
|  | 1 time a week | 468 | 12.39 |  |  | 0.0185 (0.0172, 0.0199) |  |  |  | 0.0183 (0.0171, 0.0197) |
|  | 2 times a week | 501 | 16.17 |  |  | 0.0178 (0.0165, 0.0191) |  |  |  | 0.0177 (0.0165, 0.0190) |
|  | >2 time a week | 541 | 18.30 |  |  | 0.0170 (0.0158, 0.0182) |  |  |  | 0.0170 (0.0159, 0.0181) |
| PFHxS | never | 418 | 7.18 | 0.8530 |  | 0.9974 (0.8916, 1.1157) |  |  |  |  |
|  | 1 time a week | 471 | 2.76 |  |  | 1.0358 (0.9413, 1.1399) |  |  |  |  |
|  | 2 times a week | 502 | 3.39 |  |  | 1.0290 (0.9417, 1.1244) |  |  |  |  |
|  | >2 time a week | 543 | 3.68 |  |  | 1.0465 (0.9577, 1.1436) |  |  |  |  |
| PFOA | never | 418 | 0.00 | 0.9326 |  | 1.6444 (1.5212, 1.7776) |  |  |  |  |
|  | 1 time a week | 471 | 0.00 |  |  | 1.6603 (1.5521, 1.7759) |  |  |  |  |
|  | 2 times a week | 502 | 0.40 |  |  | 1.6555 (1.5404, 1.7792) |  |  |  |  |
|  | >2 time a week | 543 | 0.18 |  |  | 1.6240 (1.5206, 1.7343) |  |  |  |  |
| PFOS | never | 418 | 0.00 | 0.0298 | A | 4.3828 (4.0564, 4.7354) |  |  |  |  |
|  | 1 time a week | 471 | 0.21 |  | A | 4.7781 (4.4471, 5.1338) |  |  |  |  |
|  | 2 times a week | 502 | 0.40 |  | A | 4.7116 (4.4115, 5.0322) |  |  |  |  |
|  | >2 time a week | 543 | 0.00 |  | A | 4.3896 (4.1153, 4.6823) |  |  |  |  |

^*^ 95% CI: the 95% confidence intervals for the geometric mean were corrected for multiple comparisons using Bonferroni correction

^**^ The analysis is based on the non-parametric Wilcoxon test and Kaplan-Meier estimates of the median. Confidence intervals were calculated using the Greenwood variance estimate.

# Table S17: Results for MIREC persistent organic pollutants in maternal blood by intake of steak

| Contaminant | Group | N | %<LOD | Unadjusted | | | Lipid as a covariate | | | |
| --- | --- | --- | --- | --- | --- | --- | --- | --- | --- | --- |
|  |  |  |  | P-value | GM* (95% CI) | | P-value for lipids | P-value | GM (95% CI) | |
| Beta-HCH | never | 1312 | 32.09 | 0.8326 |  | 0.0141 (0.0128, 0.0154) | <0.0001 | 0.8154 |  | 0.0142 (0.0130, 0.0154) |
|  | 1 time a week | 383 | 31.85 |  |  | 0.0144 (0.0125, 0.0166) |  |  |  | 0.0138 (0.0118, 0.0161) |
|  | 2 times a week | 198 | 31.31 |  |  | 0.0150 (0.0122, 0.0185) |  |  |  | 0.0148 (0.0119, 0.0183) |
| DDE | never | 1337 | 1.20 | 0.2677 |  | 0.3457 (0.3272, 0.3653) | <0.0001 | 0.2956 |  | 0.3451 (0.3273, 0.3638) |
|  | 1 time a week | 392 | 0.51 |  |  | 0.3238 (0.2972, 0.3529) |  |  |  | 0.3253 (0.2950, 0.3586) |
|  | 2 times a week | 200 | 1.00 |  |  | 0.3579 (0.3107, 0.4122) |  |  |  | 0.3596 (0.3136, 0.4123) |
| Oxychlordane | never | 1337 | 7.85 | 0.4224 |  | 0.0122 (0.0117, 0.0126) | <0.0001 | 0.2420 |  | 0.0121 (0.0117, 0.0126) |
|  | 1 time a week | 390 | 6.92 |  |  | 0.0127 (0.0119, 0.0136) |  |  |  | 0.0128 (0.0120, 0.0137) |
|  | 2 times a week | 200 | 9.50 |  |  | 0.0123 (0.0110, 0.0137) |  |  |  | 0.0124 (0.0113, 0.0136) |
| PBDE47 | never | 1331 | 33.81 | 0.8641 |  | 0.0436 (0.0405, 0.0469) | <0.0001 | 0.9374 |  | 0.0433 (0.0403, 0.0466) |
|  | 1 time a week | 391 | 35.29 |  |  | 0.0418 (0.0363, 0.0482) |  |  |  | 0.0424 (0.0371, 0.0484) |
|  | 2 times a week | 200 | 35.00 |  |  | 0.0423 (0.0348, 0.0515) |  |  |  | 0.0428 (0.0356, 0.0515) |
| PCB | never | 1337 | 2.69 | 0.3050 |  | 0.3649 (0.3482, 0.3824) | <0.0001 | 0.2554 |  | 0.3640 (0.3473, 0.3814) |
|  | 1 time a week | 392 | 1.53 |  |  | 0.3833 (0.3507, 0.4188) |  |  |  | 0.3853 (0.3534, 0.4200) |
|  | 2 times a week | 200 | 5.00 |  |  | 0.3494 (0.3070, 0.3977) |  |  |  | 0.3515 (0.3114, 0.3967) |
| PCB118 | never | 1337 | 26.10 | 0.2766 |  | 0.0146 (0.0140, 0.0153) | <0.0001 | 0.3120 |  | 0.0146 (0.0140, 0.0152) |
|  | 1 time a week | 392 | 28.06 |  |  | 0.0144 (0.0133, 0.0157) |  |  |  | 0.0145 (0.0134, 0.0157) |
|  | 2 times a week | 200 | 28.00 |  |  | 0.0136 (0.0122, 0.0152) |  |  |  | 0.0135 (0.0121, 0.0151) |
| PCB138 | never | 1337 | 6.81 | 0.4242 |  | 0.0257 (0.0245, 0.0269) | <0.0001 | 0.3613 |  | 0.0256 (0.0245, 0.0268) |
|  | 1 time a week | 392 | 6.89 |  |  | 0.0267 (0.0244, 0.0292) |  |  |  | 0.0269 (0.0247, 0.0292) |
|  | 2 times a week | 200 | 9.00 |  |  | 0.0247 (0.0218, 0.0280) |  |  |  | 0.0248 (0.0221, 0.0280) |
| PCB153 | never | 1337 | 1.27 | 0.3180 |  | 0.0444 (0.0423, 0.0465) | <0.0001 | 0.2713 |  | 0.0443 (0.0422, 0.0464) |
|  | 1 time a week | 392 | 0.77 |  |  | 0.0467 (0.0426, 0.0511) |  |  |  | 0.0469 (0.0429, 0.0512) |
|  | 2 times a week | 200 | 2.50 |  |  | 0.0426 (0.0373, 0.0486) |  |  |  | 0.0428 (0.0378, 0.0484) |
| PCB170++ | never | 1337 | 47.12 | 0.3088 |  | 0.0102 (0.0095, 0.0109) | <0.0001 | 0.2645 |  | 0.0101 (0.0095, 0.0108) |
|  | 1 time a week | 392 | 44.39 |  |  | 0.0110 (0.0097, 0.0125) |  |  |  | 0.0110 (0.0098, 0.0124) |
|  | 2 times a week | 200 | 50.50 |  |  | 0.0097 (0.0079, 0.0118) |  |  |  | 0.0099 (0.0084, 0.0117) |
| PCB180 | never | 1337 | 6.81 | 0.3354 |  | 0.0297 (0.0282, 0.0313) | <0.0001 | 0.3103 |  | 0.0296 (0.0281, 0.0313) |
|  | 1 time a week | 392 | 8.16 |  |  | 0.0311 (0.0281, 0.0345) |  |  |  | 0.0313 (0.0283, 0.0345) |
|  | 2 times a week | 200 | 10.00 |  |  | 0.0280 (0.0241, 0.0325) |  |  |  | 0.0282 (0.0246, 0.0324) |
| Transnonachlor | never | 1337 | 16.08 | 0.7767 |  | 0.0177 (0.0171, 0.0185) | <0.0001 | 0.6637 |  | 0.0177 (0.0170, 0.0184) |
|  | 1 time a week | 391 | 15.09 |  |  | 0.0181 (0.0168, 0.0195) |  |  |  | 0.0182 (0.0170, 0.0196) |
|  | 2 times a week | 200 | 16.50 |  |  | 0.0174 (0.0154, 0.0195) |  |  |  | 0.0176 (0.0159, 0.0195) |
| PFHxS | never | 1341 | 4.03 | 0.0239** | A | 1.0000 (0.9316, 1.0684) |  |  |  |  |
|  | 1 time a week | 391 | 4.35 |  | A | 1.0000 (0.8917, 1.1083) |  |  |  |  |
|  | 2 times a week | 202 | 4.46 |  | A | 0.8700 (0.7495, 0.9905) |  |  |  |  |
| PFOA | never | 1341 | 0.15 | 0.1035 |  | 1.6703 (1.6074, 1.7356) |  |  |  |  |
|  | 1 time a week | 391 | 0.26 |  |  | 1.6264 (1.5155, 1.7454) |  |  |  |  |
|  | 2 times a week | 202 | 0.00 |  |  | 1.5229 (1.3776, 1.6835) |  |  |  |  |
| PFOS | never | 1341 | 0.15 | 0.1734 |  | 4.5873 (4.4154, 4.7658) |  |  |  |  |
|  | 1 time a week | 391 | 0.26 |  |  | 4.6463 (4.3250, 4.9914) |  |  |  |  |
|  | 2 times a week | 202 | 0.00 |  |  | 4.2511 (3.8925, 4.6428) |  |  |  |  |

^*^ 95% CI: the 95% confidence intervals for the geometric mean were corrected for multiple comparisons using Bonferroni correction

# Table S18: Comparison of demographic groups when the INTERACTION between BMI and total lipid was significant

| contaminant | Pre-BMI | P25 of total lipid | P50 of total lipid | P75 of total lipid |
| --- | --- | --- | --- | --- |
|  |  | Adjusted GM (95% CI) | Adjusted GM (95% CI) | Adjusted GM (95% CI) |
| Beta-HCH | Underweight to Normal (BMI < 25) | 0.0117 (0.0105, 0.0130) | 0.0141 (0.0128, 0.0154) | 0.0174 (0.0156, 0.0193) |
|  | Overweight (25 <= BMI < 30) | 0.0130 (0.0107, 0.0158) | 0.0144 (0.0123, 0.0168) | 0.0161 (0.0137, 0.0189) |
|  | Obese (BMI >=30) | 0.0135 (0.0104, 0.0176) | 0.0148 (0.0120, 0.0183) | 0.0165 (0.0137, 0.0199) |
| DDE | Underweight to Normal (BMI < 25) | 0.3274 (0.3074, 0.3488) | 0.3641 (0.3443, 0.3851) | 0.4112 (0.3840, 0.4403) |
|  | Overweight (25 <= BMI < 30) | 0.3165 (0.2806, 0.3571) | 0.3296 (0.2990, 0.3633) | 0.3452 (0.3122, 0.3817) |
|  | Obese (BMI >=30) | 0.2792 (0.2369, 0.3290) | 0.2897 (0.2544, 0.3299) | 0.3022 (0.2690, 0.3396) |

P25 of total lipid is the 25^th^ percentile of total lipid (5.4 g/L); P50 of total lipid is the 50^th^ percentile of total lipid (6.1 g/L); P75 of total lipid is the 75^th^ percentile of total lipid (6.9 g/L)

# Table S19: Comparison of demographic groups when the INTERACTION was significant between year of collection and total lipid

| contaminant | Group | P25 of total lipid | P50 of total lipid | P75 of total lipid |
| --- | --- | --- | --- | --- |
|  |  | Adjusted GM (95% CI) | Adjusted GM (95% CI) | Adjusted GM (95% CI) |
| DDE | 2008 | 0.3454 (0.2820, 0.4231) | 0.3674 (0.3089, 0.4369) | 0.3942 (0.3147, 0.4938) |
|  | 2009 | 0.3562 (0.3249, 0.3905) | 0.3649 (0.3373, 0.3947) | 0.3751 (0.3455, 0.4072) |
|  | 2010 | 0.2974 (0.2736, 0.3232) | 0.3288 (0.3069, 0.3522) | 0.3687 (0.3404, 0.3994) |
|  | 2011 | 0.2092 (0.1625, 0.2693) | 0.2367 (0.1905, 0.2941) | 0.2726 (0.2081, 0.3570) |
| PBDE47 | 2008 | 0.0407 (0.0307, 0.0538) | 0.0520 (0.0413, 0.0655) | 0.0688 (0.0513, 0.0924) |
|  | 2009 | 0.0432 (0.0382, 0.0489) | 0.0457 (0.0411, 0.0508) | 0.0487 (0.0437, 0.0544) |
|  | 2010 | 0.0343 (0.0305, 0.0386) | 0.0388 (0.0352, 0.0427) | 0.0446 (0.0400, 0.0497) |
|  | 2011 | 0.0416 (0.0295, 0.0587) | 0.0408 (0.0304, 0.0548) | 0.0399 (0.0276, 0.0577) |

# Table S20: Comparison of demographic groups when the INTERACTION was significant between intake of bacon and total lipid

| contaminant | Group | P25 of total lipid | P50 of total lipid | P75 of total lipid |
| --- | --- | --- | --- | --- |
|  |  | Adjusted GM (95% CI) | Adjusted GM (95% CI) | Adjusted GM (95% CI) |
| DDE | never | **0.3350** (0.3130, 0.3586) | **0.3506** (0.3307, 0.3717) | **0.3693** (0.3461, 0.3941) |
|  | 1 time a week | 0.2831 (0.2544, 0.3149) | 0.3143 (0.2878, 0.3433) | 0.3543 (0.3213, 0.3906) |
|  | 2 times a week | 0.3066 (0.2691, 0.3493) | 0.3302 (0.2969, 0.3673) | 0.3595 (0.3192, 0.4049) |
| PBDE47 | never | **0.0397** (0.0361, 0.0436) | **0.0420** (0.0388, 0.0455) | 0.0448 (0.0410, 0.0489) |
|  | 1 time a week | 0.0381 (0.0330, 0.0441) | 0.0443 (0.0393, 0.0499) | **0.0525 (**0.0462, 0.0597) |
|  | 2 times a week | 0.0353 (0.0295, 0.0423) | 0.0401 (0.0347, 0.0464) | 0.0465 (0.0396, 0.0544) |

# Table S21: Results for MIREC persistent organic pollutants in cord blood by infant gender (µg/L)

| contaminant | Gender | P-value | GM (95% CI) | |
| --- | --- | --- | --- | --- |
| PFOA | Male | 0.4737 |  | 0.3536 (0.3336, 0.3747) |
|  | Female |  |  | 0.3428 (0.3224, 0.3646) |

# Table S22: Results for MIREC persistent organic pollutants in cord blood by season of collection (µg/L)

| **contaminant** | **season of collection** | **P-value** | **GM (95% CI)** | |
| --- | --- | --- | --- | --- |
| PFOA | fall | 0.0840 |  | 0.3676 (0.3275, 0.4127) |
|  | spring |  |  | 0.3361 (0.3019, 0.3742) |
|  | summer |  |  | 0.3684 (0.3294, 0.4121) |
|  | winter |  |  | 0.3235 (0.2867, 0.3650) |

**Table S23: Results for MIREC persistent organic pollutants in cord blood by smoking status of mother (µg/L)**

| **contaminant** | **smoking status** | **P-value** | **GM (95% CI)** | |
| --- | --- | --- | --- | --- |
| PFOA | Current | 0.1282 |  | 0.3802 (0.3253, 0.4445) |
|  | Former |  |  | 0.3652 (0.3303, 0.4039) |
|  | Never |  |  | 0.3384 (0.3166, 0.3618) |
